# Mitolnc controls cardiac BCAA metabolism and heart hypertrophy by allosteric activation of BCKDH

**DOI:** 10.1093/nar/gkae226

**Published:** 2024-04-03

**Authors:** Maria Weiss, Sara Hettrich, Theresa Hofmann, Salma Hachim, Stefan Günther, Thomas Braun, Thomas Boettger

**Affiliations:** Max Planck Institute for Heart- and Lung Research, Department of Cardiac Development and Remodelling, Ludwigstr. 43, D-61231 Bad Nauheim, Germany; Max Planck Institute for Heart- and Lung Research, Department of Cardiac Development and Remodelling, Ludwigstr. 43, D-61231 Bad Nauheim, Germany; Max Planck Institute for Heart- and Lung Research, Department of Cardiac Development and Remodelling, Ludwigstr. 43, D-61231 Bad Nauheim, Germany; Max Planck Institute for Heart- and Lung Research, Department of Cardiac Development and Remodelling, Ludwigstr. 43, D-61231 Bad Nauheim, Germany; Max Planck Institute for Heart- and Lung Research, Department of Cardiac Development and Remodelling, Ludwigstr. 43, D-61231 Bad Nauheim, Germany; Max Planck Institute for Heart- and Lung Research, Department of Cardiac Development and Remodelling, Ludwigstr. 43, D-61231 Bad Nauheim, Germany; Max Planck Institute for Heart- and Lung Research, Department of Cardiac Development and Remodelling, Ludwigstr. 43, D-61231 Bad Nauheim, Germany

## Abstract

Enzyme activity is determined by various different mechanisms, including posttranslational modifications and allosteric regulation. Allosteric activators are often metabolites but other molecules serve similar functions. So far, examples of long non-coding RNAs (lncRNAs) acting as allosteric activators of enzyme activity are missing. Here, we describe the function of mitolnc in cardiomyocytes, a nuclear encoded long non-coding RNA, located in mitochondria and directly interacting with the branched-chain ketoacid dehydrogenase (BCKDH) complex to increase its activity. The BCKDH complex is critical for branched-chain amino acid catabolism (BCAAs). Inactivation of mitolnc in mice reduces BCKDH complex activity, resulting in accumulation of BCAAs in the heart and cardiac hypertrophy via enhanced mTOR signaling. We found that mitolnc allosterically activates the BCKDH complex, independent of phosphorylation. Mitolnc-mediated regulation of the BCKDH complex constitutes an important additional layer to regulate the BCKDH complex in a tissue-specific manner, evading direct coupling of BCAA metabolism to ACLY-dependent lipogenesis.

## Graphical Abstract

**Graphical Abstract:**
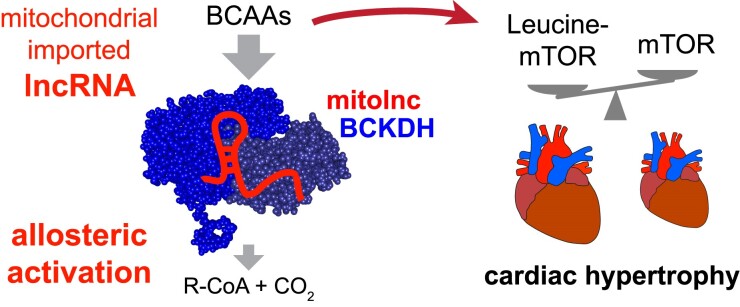

## Introduction

The essential branched chain amino acids (BCAAs) valine, leucine and isoleucine are important building blocks of life, contributing to protein synthesis. Moreover, BCAAs and some of their products serve as signaling molecules, regulating protein synthesis, insulin secretion and other processes. Delicate changes in BCAA metabolism are assumed to contribute to heart failure and several other widespread diseases (1). The first steps of BCAA catabolism are common for all three BCAAs and consist of a reversible transamination and a virtually irreversible oxidative decarboxylation. Reversible transamination is achieved by BCAA aminotransferases (BCATs) that transfer the amino group of BCAAs to α-ketoglutarate, generating branched-chain ketoacids (BCKAs). BCAT1 is localized in the cytosol and BCAT2 is localized at the inner membrane of the mitochondria where it forms a temporary structural-functional complex, a metabolon, with the branched chain ketoacid dehydrogenase complex (BCKDH complex), which mediates the second, rate-limiting step of irreversible oxidative decarboxylation. The BCKDH complex consists of the three components E1, E2 and E3. The E1 component is a thiamine-dependent decarboxylase, containing BCKDHA and BCKDHB heterotetramers. The E2 component is DBT, a dihydrolipoyl transacylase mediating the transfer of the acyl group to coenzyme A. The E3 component is DLD, an FAD-dependent dihydrolipoyl dehydrogenase mediating the transfer of electrons to NAD^+^. Subsequent processing of the branched chain acyl-CoAs diverges according to the structure of the acyl group (1). Mutation of the BCKDH complex subunits result in maple syrup urine disease (MSUD), characterized by toxic levels of free BCAAs and their corresponding BCKAs. MSUD may result in ketoacidosis and severe neurological disease and regularly leads to death if left untreated (2,3).

Oxidative decarboxylation by the BCKDH complex is tightly controlled by phosphorylation of the E1 subunit BCKDHA at serine 293. Phosphorylation by BCKDH kinase (BDK) (4) results in inactivation of the BCKDH complex while PPM1K is the corresponding phosphatase activating the BCKDH complex (5,6). BDK is allosterically inhibited by branched-chain ketoacids, creating a positive feedback loop. Interestingly, BDK and PPM1K also have essential roles in lipid metabolism by regulating the activity of ATP-citrate lyase (ACLY), which catalyzes the conversion of citrate to acetyl-CoA and oxaloacetate, thereby promoting lipogenesis and reducing fatty acid oxidation (7,8). BDK activates ACLY by phosphorylation at serine 454 and dephosphorylation by PPM1K inhibits ACLY, integrating BCAAs catabolism with lipogenesis (9,10).

The BCAA leucine has well described anabolic effects (11), achieved by stimulation of mTOR signaling via different mechanisms. Leucine is sensed by Sestrin1/2 and activates mTOR signals via GATOR2/1-RAG (12,13). Leucine might also be sensed by the SLC38A9-RAG-Ragulator complex at the lysosomal surface to activate mTOR via Rheb (14,15). Alternatively, leucine concentration might also be detected by the leucyl tRNA synthetase 1 (LARS1) (16), whose activity is glucose-dependent (17), illustrating the complexity of regulatory networks involving leucine. Another important signaling effect of leucine is the allosteric activation of glutamate dehydrogenase (GDH) (18,19), yielding α-ketoglutarate.

Long non-coding RNAs (lncRNAs) have not been assumed to play a direct role in the regulation of BCAA metabolism so far. LncRNAs are longer than 200 nucleotides, are not translated into functional proteins, usually show a very poor conservation of their primary sequences during evolution, and are generated by widespread transcription of genomes, resulting in a highly heterogeneous group of transcripts with different biogenesis and genomic origin. Current estimates range from 16 000 to 100 000 lncRNA genes in the human genome (20,21). An increasing number of functional studies indicate the importance of lncRNAs for the regulation of multiple cellular processes. In many cases, lncRNAs regulate chromatin activity, often *in cis*, next to lncRNA-expressing loci (22,23) or *in trans* within the nucleus to activate and repress gene activity (24). Moreover, several lncRNAs are exported to the cytosol, probably sharing the same pathways with mRNAs. Some lncRNAs are sorted into mitochondria such as the RNA component of mitochondrial RNA-processing endoribonuclease (RMRP), which is bound and stabilized by the G-rich RNA sequence-binding factor 1 (GRSF1), enabling enrichment in the mitochondrial matrix (25). However, only limited insight exists regarding the physiological function of lncRNAs, which are limited to *in vitro* studies in melanoma cells, fibroblasts and macrophages (25–29).

In this study, we investigate the physiological roles of cardiac-restricted and nucleus-encoded lncRNAs that are transferred into mitochondria. We identified a lncRNA (AK079912) that we named mitolnc, which interacts with the BCKDH complex in the mitochondrial matrix and allosterically regulates its activity. Inactivation of mitolnc reduces activity of the BCKDH complex and results in accumulation of BCAAs in the adult heart, which enhances mTOR signaling and induces cardiac hypertrophy. Mitolnc-dependent control of the BCKDH complex circumvents phosphorylation-based regulation by BDK and PPM1K, thereby uncoupling BCAA catabolism from fatty acid metabolism in the heart.

## Materials and methods

### Animal models

Two pairs of sgRNAs were used in a double nicking approach (30) to direct hSpCas9n (D10A) expressed from a modified pX335 vector (addgene #42335) carrying an additional EF1a-copGFP-T2A-Puromycin^R^ cassette. Pairs of sgRNAs were designed to target sequences 5′ (guide sequences: p894: GGATGAAATTTACTTTAAGT, p895: gCCATGCAAGAGAAAACTCAT) and 3′ (p898: GTGCACAATAGTTTTGGTTT, p899: GTACCTAAAGCCCTGGGTGA) of the genomic locus of AK079912. The four px335-derived plasmids containing the respective U6-driven sgRNAs were transfected into mouse embryonic stem cells using Lipofectamine™ 3000 (Thermo Fisher #L3000001) followed by puromycin selection (2 μg/μl, 1 day after transfection for 2 days) to obtain mutant clones that were detected by PCR using oligonucleotides. All oligonucleotide sequences used in this study are listed in Supplementary Table S1.

For an alternative loss of function model, a poly A site was inserted into the first exon of AK079912 using the px459 pSpCas9(BB)-2A-Puro vector (pSpCas9(BB)-2A-Puro (PX459) V2.0 was a gift from Feng Zhang (Addgene plasmid #62988; http://n2t.net/addgene:62988;RRID:Addgene_62988) (31). The px459 vector was used with a specific guide sequence (gCAGAGCTTGGGATCCCTCAT) and in combination with a single-stranded oligo (cattgaggatcctggctctgctaaggactctgcactctgcttagaatcctggctccaatgagggatcccaaattctggttacaaataaagcaatagcatcacaaatttcacaaataaagcatttttttcacctggctctgacatctacttccagtgaccttggacaagttgctccttccttctagagctcagtcttccaa) for homology directed repair. 0.5 μg of the plasmid and 1.5 μg of oligonucleotide were transfected into 3 × 10^5^ embryonic stem cells using Lipofectamine™ 3000, followed by puromycin selection (2 μg/μl, 48 h). Clones with recombinant alleles were identified by PCR using oligonucleotides listed in Supplementary Table S1. Amplicons were sequenced to identify correctly targeted clones. Chimeric mice were generated by injection of embryonic stem cells into blastocysts, chimeras were backcrossed to 129SV mice and heterozygous mice were mated to obtain mitolnc^−/−^ or mitolnc^pA/pA^ mice and the respective WT mice. High BCAA diet was obtained from Sniff (www.sniff.com, S7507-E054, 4.3% BCAA; Crystalline AA diet). Mice were fed ad libitum for 8 weeks. Encapsulated Rapamycin was obtained from Rapamycin Holdings (www.rapamycinholdings.com) to produce 14 ppm active rapamycin supplemented diet and respective control diet containing the rapamycin vehicle Eudragit by Envigo—Teklad Diets (TD.160060 based on 2018 Teklad Global 18% Protein Rodent Diet, www.envigo.com). Mice were fed ad libitum for 8 weeks. If not otherwise stated mice were kept in IVC with ad libitum access to water and food. All animal experiments were done in accordance with national and European community guidelines and were approved by the Committee for Animal Protection of the State of Hessen (Regierungspraesidium Darmstadt).

### BCAA serum concentration

To analyze BCAA levels in blood serum we sacrificed the mice via decapitation and collected the blood. To obtain blood serum the blood coagulated for 5 min at room temperature followed by 5 min centrifugation at 14 000 rpm. BCAA levels of serum samples were obtained by using the branched chain amino acid assay kit from abcam (#ab83374) according to manufacturer's specifications.

### Isolation of mouse cardiomyocytes

Animals were sacrificed via i.p. injections of 100 μl/10 g bodyweight Ketamin/Xylariem (4 ml NaCl 0.9%, 0.5 ml Ketamin, 0.25 ml Xylariem). Hearts were collected and washed and perfused with perfusion buffer (NaCl 113 mM, KCl 4.7 mM, Na_2_HPO_4_ 0.6 mM, KH_2_PO_4_ 0.6 mM, MgSO_4_ 1.2 mM, NaHCO_3_ 12 mM, KHCO_3_ 10 mM, taurine 30 mM, HEPES 10 mM, 2,3-butanedione monoxime 10 mM, glucose 5.5 mM). Cells were digested via perfusion of the hearts with enzyme buffer (perfusion buffer, Liberase DH 0.25 mg/ml (Roche #05401089001), Trypsin 0.27 mg/ml (Sigma #T7409), CaCl_2_ 23.2 μM). The enzymatic reaction was stopped by adding Stop buffer (perfusion buffer, FCS 5%, CaCl_2_ 12.5 μM).

### Isolation of nuclei and cytoplasm

For isolation of nuclei and cytoplasm, mouse cardiomyocytes were isolated and washed with ice-cold PBS pH 7.4. Samples were centrifuged (1000 × g, 10 min at 4°C) and the pellet re-suspended in 100 μl Buffer A (10 mM Tris/HCl pH 8.0, 140 mM NaCl, 1.5 mM MgCl_2_, 0.5% Nonidet P-40). Samples were incubated on ice for 5 min and then centrifuged (1000 × g, 3 min at 4°C). The supernatant, containing the extranuclear fraction was collected and mixed with 700 μl Trizol for RNA isolation. The pellet was washed twice using Buffer A and then re-suspended in Buffer B (Buffer A + 1% Tween-40, 0.5% sodium deoxycholate) followed by another centrifugation for 5 min. The pellet containing the nuclear fraction was re-suspended in 700 μl Trizol for RNA isolation.

### Isolation of mitochondria

Mitochondrial isolation was performed as described previously (32,33). In brief, mice were sacrificed by cervical dislocation and perfused with PBS. Hearts were transferred into PBD (1 × PBS pH 7.4, 10 mM EDTA) and minced using a pair of scissors. The tissue was washed twice with PBD and then incubated with PBD-T (PBD, 0.05% Trypsin) on ice for 30 min. The samples were centrifuged at 200 × g for 5 min at 4°C. The pellet was resuspended in 5 ml IBM_1_ pH 7.4 (6.7 ml Sucrose (1 M), 5 ml Tris/HCl (1 M), 5 ml KCl (1 M), 2 ml BSA (10%) ad. 100 ml ddH_2_O). Samples were homogenized using a potter homogenizer (10 strokes at 1600 rpm). The homogenate was centrifuged (700 × g, 10 min at 4°C). The pellet (cell debris) was used as heart/rest fraction in the downstream analysis. The supernatant containing the mitochondria was transferred into glass centrifugation tubes followed by centrifugation (8000 × g, 10 min at 4°C). The supernatant was removed, and the pellet was re-suspended in 5 ml IBM_2_ pH 7.4 (25 ml Sucrose (1 M), 3 ml EGTA/Tris (1 M), 1 ml Tris/HCl (1 M) ad. 100 ml ddH_2_O) followed by centrifugation (8000 × g, 10 min at 4°C) and resuspension of the pellet in an equal volume of IBM_2_.

For RNA isolation, samples were further treated with 1 μl Digitonin (Sigma #D141, 10 mg/ml) and 0.5 μl RNase A (Merck #20297, 10 mg/ml). Samples were incubated for 20 min at 27°C. Afterwards samples were centrifuged at 11 000 × g for 5 min at 4°C. The pellet containing the mitochondria was re-suspended in 700 μl TRIzol and RNA isolation was performed using RNeasy Mini Kit (Qiagen #217004) combined with on-column DNase digestion (RNase-Free DNase Set, Qiagen #79254) according to manufacturer's instruction.

### Quantification of cardiomyocytes cross sectional area

Cryosections of heart tissue were used to determine cardiomyocytes cross sectional area. Hearts were collected after cervical dislocation of animals, were fixed using 4% PFA/PBS (2 h, 4°C) and transferred to 15% sucrose/PBS, 30% sucrose/PBS and Tissue-Tek® O.C.T Compound (94-4583-1; each step overnight, 4°C). Cryosections (10 μm) were prepared using a Leica CM1950, dried (30 min, 21°C) and fixed (4% PFA, 10 min, 21°C). Slides were incubated in 0.1% Triton X-100/PBS (10 min, 21°C) and washed three times using PBS (5 min each). Samples were stained using wheat germ agglutinin (WGA-488, Thermo #W11261, 1 mg/ml, 1:500 in carrier containing 5% goat serum, 2% BSA, 0.1% Triton X-100 in PBS; 1 h, 21°C). Sections were incubated using DAPI (Invitrogen #D3571, 2 mg/ml, 1:1000 in PBS, 5 min 21°C), washed three times (PBS, 5 min each), embedded using Fluoromount (Sigma #F4680) and microscopic images were acquired using an Axio Observer (Zeiss). Cross sectional area of cardiomyocytes was determined using blinded samples and manual segmentation in ImageJ. Numerical binning of cross sectional area was used for statistical analysis.

### RNA seq, Affymetrix microarrays and RT-qPCR

For RNA-seq, RNA was isolated from isolated mitochondria using TRIzol™ (Thermo Scientific #15596026) according to the manufacturer's instruction, followed by miRNeasy mini Kit (Qiagen #217004) combined with on-column DNase digestion (RNase-Free DNase Set, Qiagen #79254). RNA and library preparation integrity were verified with LabChip Gx Touch 24 (Perkin Elmer). 10 ng of total RNA was used as input for SMARTer® Stranded Total RNA-Seq Kit—Pico Input Mammalian (Takara Clontech). Fragmentation time of mitochondrial samples was adjusted to 1 min according to size of RNA. Sequencing was performed using the NextSeq500 instrument (Illumina) using v2 chemistry, generating a minimum of 40M reads per library with 1 × 75 bp single end setup. The resulting raw reads were assessed for quality, adapter content and duplication rates with FastQC (http://www.bioinformatics.babraham.ac.uk/projects/fastqc). Trimmomatic version 0.39 was employed to trim reads after a quality drop below a mean of Q20 in a window of 5 nucleotides (34). Only reads between 30 and 150 nucleotides were cleared for further analyses. Trimmed and filtered reads were aligned versus the Ensembl mouse genome version mm10 (GRCm38.p5) using STAR 2.7.3a with the parameter ‘–outFilterMismatchNoverLmax 0.1’ to increase the maximum ratio of mismatches to mapped length to 10% (35). The number of reads aligning to genes was counted with featureCounts tool from the Subread package 1.6.5 (36). Only reads mapping at least partially inside exons were admitted and aggregated per gene. Reads overlapping multiple genes or aligning to multiple regions were excluded. Differentially expressed genes were identified using DESeq2 version 1.26.0 (37). Fold change values and Benjamini–Hochberg corrected *P*-values were used in a volcano plot. The ENCODE dataset (38) ENCSR247RPX (doi:10.17989/ENCSR247RPX) was re-evaluated for analysis of developmental expression of mitolnc.

For microarray analysis, RNA was isolated using the TRIzol method. RNA labelling and hybridization to Affymetrix MTA-1.0 arrays was performed using the GeneChip WT Plus Reagent Kit as per manufacturer's instructions. Data were analyzed using DNAstar ArrayStar with RMA and evaluated using moderated *t*-test. Gene set enrichment analysis (GSEA) was performed using the GSEA desktop software application with Molecular Signatures Database 7.0 (http://www.gsea-msigdb.org/gsea/index.jsp) with default parameters but using the permutation type gene set. GSEA determines whether a predefined set of genes shows statistically significant, concordant differences between two biological states and thus helps to identify relevant biological processes (39).

RNA for RT-qPCR and TaqMan Gene Expression Assays was isolated from tissue or cells using the TRIzol method. cDNA was synthesized using 1 μg of RNA (200 ng of mitochondrial RNA) in combination with the PrimeScript RT-PCR Kit (Takara #RR014B) according to manufacturer's instructions. For qPCR we used the Biozym Blue S′Green qPCR KIT separate ROX (#331416S) and the StepOne™ Real-Time PCR System (Thermo). Sequences of oligonucleotide used in this study are listed in Supplementary Table S1. Relative expression was determined using the ΔΔCt calculation. Alternatively predesigned TaqMan gene expression assays (Thermo) were used (FAM-labeled: BCKDHA #Mm00476112_m1; BCKDHB #Mm01177077_m1; DBT #Mm00501651_m1; DLD #Mm00432831_m1; BDK #Mm00437777_m1; PPM1K #Mm00615792_m1; ACLY #Mm01302282_m1, GAPDH #Mm999999915_g1 (VIC-labelled, PL)).

### RNA-FISH

RNA-FISH was performed using isolated cardiomyocytes as previously described (40) using ViewRNA ISH Cell Assay Kit (Affymetrix, #QVC0001) with the probe sets detecting mitolnc (#VB1-18013, type1-Alexa546, custom-designed against bases 21–266 of AK079912) and ATP6 (#VB4-3113886, type4-Alexa488). The experiment was performed according to manufactures instructions on isolated mouse cardiomyocytes which were fixed using 4% PFA/PBS for 10 min at room temperature.

### RNA–protein pull-down

For RNA–protein pull-down experiments mitolnc and control lncRNAs were *in vitro* transcribed using appropriate DNA Templates. Templates for RNA synthesis were prepared either by digestion of plasmid vectors (MS-based analysis: AK086006, Fantom2-cloneID: D830044D09, BamHI [ctrl I]; AK010044, Fantom2-cloneID: 2310065F04, ScaI [ctrl II]; western blot based analysis: AK013794, Fantom2-cloneID: 2900076A07, Ecl136II [ctrl]) or by PCR using a plasmid (AK079912, Fantom2-cloneID: A530001O10 [mitolnc]) with appropriate oligonucleotides also providing a T7 RNA polymerase promoter (Supplementary Table S1). *In vitro* transcription was performed using the T7 RiboMAXX™ Express Large Scale RNA Production System (Promega #P1320) according to the supplier's protocol, but with extension of transcription time to 2 h. For biotinylation of the RNA we used Pierce™ RNA 3′End Desthiobiotinylation Kit (Thermo Scientific #20163) according to manufacturer's instructions. RNA-pulldown was performed using the Pierce^TM^ Magnetic RNA-Protein Pull-Down Kit (Thermo Scientific #20164). For protein extracts of heart tissue, hearts of wildtype mice were chopped in a mortar on dry ice and sonicated in 1 ml RIPA buffer (50 mM Tris–HCl pH 7.4, 1% NP-40, 0.25% sodium deoxycholate, 150 mM NaCl, 1x Protease Inhibitor Cocktail III [Merck #539134], 1 U RNasin [Promega #N2611]). The beads were washed twice with RIPA buffer followed by centrifugation (12 500 rpm, 30 s, 4°C). Interacting proteins were identified by mass spectrometry after off-bead digestion as previously described (40). Additionally, RNA–protein pull-down was performed using *in vitro* transcribed RNA and protein extracts of HEK293 cells overexpressing the respective proteins V5 tagged at the C-terminus in combination with western blot analysis.

### Identification of RNA–protein interaction sites

RNA–protein interaction sites were identified following the protocol of Bae *et al.* (41). Mitochondria were isolated from single hearts as described previously and resuspended in 200 μl storage buffer. The suspension was exposed to UV irradiation at 0.150 J/cm^2^ in a 12-well plate, followed by acetone precipitation in 80% acetone at –20°C for 60 min. The pellet was resuspended in 30 μl 8 M urea/thiourea (6/2 M), followed by reduction using 30 mM DTT for 30 min at room temperature. Reduced proteins were alkylated by 55 mM Iodoacetamide (IAA) for 30 min at room temperature in dark. Proteins were cleaved by 0.5 μg sequencing grade LysC (Fuji) for 3 h at room temperature. Samples were diluted to 2 M urea/thiourea using 100 mM triethylammonium bicarbonate (TEAB) and subsequently digested by 0.5 μg sequencing grade trypsin (Serva) over night at room temperature. 800 μl 48% hydrofluoric acid was added per 5 μg protein, followed by 20 h incubation at room temperature. The samples were dried in a vacuum concentrator, pellets resuspended in 120 μl 0.5% TFA. Samples were desalted using stage tips and evaporated. Samples were reconstituted in 5 μl buffer A (0.1% formic acid). 3 μl of peptide mixture was injected and analyzed by LC–MS/MS method. Quantitative analysis was performed on an Orbitrap Q-exactive HF mass spectrometry system (Thermo Scientific) coupled to an EASY-nLC capillary nano-chromatography system (Thermo Scientific). Extracted peptides were separated on an in-house-made capillary column (150 mm × 1.7 μm × 75 μm) packed with ReproSil-Pur 120 C18-AQ resin (Dr. Maisch). The mobile phases were (A) 2% acetonitrile, 0.1% fomic acid (B) 90% acetonitrile, 0.1% formic acid. Peptides were separated using a 120 min acetonitrile gradient at room temperature. The mass spectrometer was operated in positive electrospray ionization (ESI) mode, and MS/MS data were collected in data dependent analysis mode with a resolution of 60 000 for precursor mass spectra and 15 000 for tandem mass spectra. Normalized collision energy was set to 28 and exclusion time was set to 30 s. Collected data were processed using MSGF+ software.

### RNA *in situ* proximity ligation assay (rISH-PLA)

The rISH-PLA experiment was performed as previously described (40,42) using a mix of probes directed against mitolnc designed by the Stellaris design tool (https://www.biosearchtech.com/support/education/stellaris-rna-fish). Sequences of oligonucleotides used in this study are listed in Supplementary Table S1. Isolated cardiomyocytes were used as previously described or incubated with medium containing 500 nM MitoTracker Deep Red FM (CST #8778) for 30 min at 37°C in 5% CO_2_ prior to fixation (4% PFA, 7 min). Antibodies used are rabbit anti-BCKDHA (Abcam #Ab90691 or #Ab126173), rabbit anti-DBT (Sigma #HPA026485), rabbit anti-CytC (CST #4280), mouse anti-biotin (Dianova, DLN-006043). Images were acquired using a Zeiss Axio Imager Z1 or a Leica SP8 confocal microscope.

### Western blot analysis

For western blot Bis–Tris SDS PAGE (ThermoFisher #NP0321BOX or #EA03752BOX for mTOR western blots) were loaded with 10 μg protein lysate per lane in 1× Laemmli buffer. Tissues were lysed in extraction buffer (0.1 M Tris/HCl pH 8.0, 0.01 M EDTA pH 8.0, 10% SDS, 1× protease inhibitor cocktail [Roche #04693132001] and PhosSTOP [Roche #04906845001] if appropriate) by sonication. Nitrocellulose membrane (GE Healthcare, Protran BA85) was used for blotting, membranes were blocked by 5% BSA/TBS-T. The following antibodies were used in 3% BSA/TBS-T: ACLY (Cell Signaling Technology #4332), ACLY-Ser455 (Cell Signaling Technology #4331), BCKDHA (Abcam #Ab90691), BCKDHA-Ser293 (Abcam #Ab200577), BDK (Santa Cruz #Sc374425), PPM1K (Sigma #Hpa023891), mTOR (Cell Signaling Technology #2983), mTOR-Ser2448 (Cell Signaling Technology #5536), 4E-BP1 (Cell Signaling Technology #9452), 4E-BP1-Thr37/46 (Cell Signaling Technology #9459), GAPDH (Cell Signaling Technology #2118), RalA (Bd #610221), V5 (Proteintech #66007-1-Ig), DBT (Sigma #Hpa026485), DLD (Thermo Scientific #Pa5-27367), MCCA (Santa Cruz #Sc-271427), HADHA (Novus Biologicals #Nbp1-83387), BCKDHB (Proteintech #1368501-AP), ß-ACTIN (Sigma #D1033), CYTC (Cell Signaling Technology #4280), PCCA (Novus Biologicals #Nbp2-32215), PD1 (Proteintech #11245–1-AP), ATP6 (Proteintech #55313-1-Ap), PCBP1 (Biorad#VPA00286), mouse IgG Isotype control (Invitrogen #10400C); anti-mouse HRP (Pierce #1858413), anti-rabbit HRP (Pierce #31460), anti-mouse IRdye800 (Rockland #611-132-122), anti-mouse Alexa680 (Invitrogen #A21057), anti-rabbit IRdye800 (Rockland #610-132-121), anti-rabbit Alexa680 (Invitrogen #A21076).

### Cell culture, transient transfection of cells

DMEM (Merck #D5796) containing 10% fetal calf serum (FCS, Sigma #F7524-500ML) and 1% penicillin–streptomycine–glutamine was used to maintain HEK293 (ATCC CRL-1573) and HepG2 (ATCC HB-9065) cells. Transient transfections of eukaryotic cells were performed using Lipofectamine™ 3000 according to manufacturer′s instructions. Incubation was performed for 24 h and cells were harvested after 48 h.

### Quantification of metabolites by ESI-LC MS/MS

For analysis of metabolite concentrations wildtype and mitolnc^−/−^ mice were sacrificed by decapitation. Blood was collected and the animals were perfused with PBS pH 7.4. Heart, liver and BAT was snap-frozen in liquid nitrogen. Blood serum was collected after coagulation (room temperature, 5 min) followed by a centrifugation at 14 000 rpm. The tissue samples were minced on dry ice using a mortar.

#### Extraction of brown fat, liver and heart for TCA metabolites analyses

Samples were lysed by adding 0.5 ml 85% methanol, followed by two freeze-thaw cycles. Next, samples were centrifuged at 15 000 × g for 5 min at room temperature. 200 μl of the clear supernatant were mixed with 10 μl 1 μg/ml internal standard mix and the samples were dried in a vacuum centrifuge (Concentrator plus, Eppendorf, Hamburg, Germany). Dried samples were reconstituted in 50 μl water containing 0.5% formic acid and finally transferred to MS glass vials ready to be analyzed.

Negative ionization ESI-LC MS/MS was performed on an Agilent 1290 Infinity LC system (Agilent, Waldbronn, Germany) coupled to a QTrap 5500 mass spectrometer (Sciex, Darmstadt, Germany). Ion source parameters were as follows: CUR 30 psi, CAD medium, ion spray voltage 4500 V, TEM 400°C, GS1 45 psi, GS2 25 psi. Reversed-phase LC separation was performed by using a Luna Omega 1.6 μm PS C18 (2.1 × 100 mm) column. Compounds were eluted with a flow rate of 0.4 ml/min and with the following 9 min gradient: 0–0.1 min 99.9% A, 0.1–4 min 94% A, 4–5 min 0.1% A, 5–6 min 0.1% A, 6–7 min 99.9% A and 7–9 min 99.9% A. Solvent A consisted of 100% water containing 0.5% formic acid and solvent B consisted of 100% methanol containing 0.5% formic acid. Column oven temperature was set to 40°C, and the autosampler was set to 4°C. Injection volume was 5 μl.

#### Extraction of brown fat, liver, heart and serum for amino acid analyses

Samples were lysed by adding 0.5 ml 85% methanol, followed by two freeze-thaw cycles. Next, samples were centrifuged at 15 000 × g for 5 min at room temperature. 200 μl of the clear supernatant was diluted 1:2 in water before continuing the extraction with the EZfaast Amino Acid Analysis Kit (phenomenex). For serum samples 100 μl per sample was used before using the EZfaast Amino Acid Analysis Kit. Extraction was done according to the manufactures protocol (https://www.phenomenex.com/Products/AminoAcidDetail/EZfaast).

Positive ionization ESI-LC MS/MS was performed on an Agilent 1290 Infinity LC system (Agilent, Waldbronn, Germany) coupled to a QTrap 5500 mass spectrometer (Sciex, Darmstadt, Germany). Ion source parameters were as follows: CUR 30 psi, CAD medium, Ion Spray Voltage 4000 V, TEM 425°C, GS1 40 psi, GS2 40 psi. Reversed-phase LC separation was performed by using an EZfaast amino acid analysis column (2 × 250 mm). Compounds were eluted with a flow rate of 0.25 ml/min and with the following 17 min gradient: 0 min 32% A, 0–13 min 17% A, 13–13.01 min 32% A and 13.01–17 min 32% A. Solvent A consisted of 100% water containing 10 mM ammonium formate and solvent B consisted of 100% methanol containing 10 mM ammonium formate. Column oven temperature was set to 40°C, and the autosampler was set to 4°C. Injection volume was 1 μl.

#### Extraction of brown fat, liver and heart for CoAs analyses

Heart and fat tissue. Samples were diluted in 0.75 ml acetonitrile/isopropanol (3:1, v/v). Next, 0.25 ml 0.1 M KH_2_PO_4_ (pH 6.7) were added, followed by 10 μl of 10 μg/ml internal standards mix. Samples were vortexed for 30 s and centrifuged at 15 000 × g for 5 min at room temperature. Clear supernatants were transferred into new vials and 0.25 ml acetic acid was added before SPE clean-up.

Liver tissue. Samples were diluted in 1.5 ml acetonitrile/isopropanol (3:1, v/v). Next, 0.5 ml 0.1 M KH_2_PO_4_ (pH 6.7) were added, followed by 10 μl of 10 μg/ml internal standards mix. Samples were vortexed for 30 s and centrifuged at 15 000 × g for 5 min at room temperature. Clear supernatants were transferred into new vials and 0.5 ml acetic acid was added before SPE clean-up (Note: internal standard mix contains labelled acetyl-CoA and palmitoyl-CoA).

SPE clean-up. SPE columns (SUPELCO 54127-U) were conditioned with 1 ml acetonitrile/isopropanol/water/acetic acid (9:3:4:4, v/v/v/v). Next, samples were loaded onto the SPE columns. After 1 min, the valves were opened allowing the samples to flow through (gravity only). Columns were washed with 1 ml acetonitrile/isopropanol/water/acetic acid (9:3:4:4, v/v/v/v) and dried under vacuum for 5 min. Sample elution was done by adding 900 μl of methanol/250 mM ammonium formate (4:1, v/v; pH 7 to ensure pyridyl-group neutralization). Next, samples were dried in a vacuum under a nitrogen stream and reconstituted in 50 μl methanol/water (1:1, v/v). Samples were vortexed and shaken at 1200 rpm for 5 min at room temperature. Last but not least, samples were centrifuged at 15 000 × g for 10 min at room temperature and finally transferred to MS glass vials ready to be analyzed.

Positive ionization ESI-LC MS/MS was performed on an Agilent 1290 Infinity LC system (Agilent, Waldbronn, Germany) coupled to a QTrap 5500 mass spectrometer (Sciex, Darmstadt, Germany). Ion source parameters were as follows: CUR 25 psi, CAD medium, Ion Spray Voltage 4500 V, TEM 500°C, GS1 40 psi, GS2 40 psi. Reversed-phase LC separation was performed using an Acquity UPLC BEH C18 1.7 μm (2.1 × 75 mm) column. Compounds were eluted with a flow rate of 0.4 ml/min and with the following 10 min gradient: 0–0.2 min 98% A, 0.2–1 min 55% A, 1–4 min 35% A, 4–4.2 min 0% A, 4.2–7 min 0% A, 7–7.1 min 98% A and 7.1–10 min 98% A. Solvent A consisted of 100% water containing 10 mM ammonium acetate with 1 ml/l Infinity Lab Deactivator Additive (pH 8.5) and solvent B consisted of acetonitrile/water (9:1, v/v) containing 10 mM ammonium acetate with 1 ml/l Infinity Lab Deactivator Additive (pH 8.5). Column oven temperature was set to 35°C, and the autosampler was set to 4°C. Injection volume was 5 μl.

### Immunoprecipitation

For immunoprecipitation 1 mg protein lysate, 30 μl G-Sepharose-beads (Sigma Aldrich #P3296) and 2 μg of the corresponding antibody were used. For protein extraction hearts were chopped in a mortar on dry ice and sonicated in 1 ml RIPA buffer (50 mM Tris–HCl pH 7.4, 1% NP-40, 0.25% sodium deoxycholate, 150 mM NaCl, 1× protease inhibitor Cocktail III (Merck #539134, 1 U RNasin [Promega #N2611]). The beads were washed twice using RIPA buffer followed by centrifugation (12 500 rpm, 30 s, 4°C). Beads were blocked with 3% BSA in RIPA Puffer (1 h, 4°C, rotating) before incubation with protein lysate and the corresponding antibody (overnight at 4°C, rotating). Samples were washed three times with RIPA buffer, re-suspended in 100 μl RIPA buffer and finally analyzed by mass spectrometry. Antibodies used were MCCA (Santa Cruz #Sc-271427), DBT (Sigma #Hpa026485) and anti-mouse IgG (Invitrogen 10400c). For western blot we used anti-mouse IgG Trueblot HRP (Ebioscience #18-8877-31) and anti-rabbit IgG Trueblot HRP (Ebioscience #18-8816-31).

### BCKDH activity assay

The BCKDH activity assay was performed as described previously (9). Animals were killed and hearts were perfused with PBS pH 7.4. The hearts were collected, weighted and washed twice with ice-cold PBS. Tissue was transferred into Buffer H (30 mM KPi monobasic pH 7.5, 3 mM EDTA, 5 mM DTT, 1 mM KIV (Sigma #198994), 3% FCS, 5% Triton X-100, 1 μM Leupeptin (Sigma #L2884) in ddH_2_O) and minced using a pair of scissors. Homogenization was performed using the gentleMACS Octo Dissociator (Miltenyi #130-096-427; C-tubes: Miltenyi #130–093-237, mouse tissue mitochondria isolation program). Samples were centrifuged at 10 000 × g, 10 min at 4°C. The assays were performed in 24-well plates, whereby 50 μl tissue lysate or 100 μl cell lysate in 300 μl Buffer A (50 mM HEPES pH 7.5, 30 mM KPi (monobasic) pH 7.5, 0.4 M CoA, 3 mM NAD^+^ (Sigma #N0632-1G), 5% FCS, 2 mM Thiamin pyrophosphate (Sigma #C8754-1G), 2 mM MgCl_2_ in ddH_2_O, and 1 μl ^14^C-KIV (ARC 3191 Ketoisovaleric acid, α-[1-^14^C] Na salt) was used. The ^14^C-labeled CO_2_ was trapped with the help of NaOH (1 M) soaked chromatin paper which was placed in the lid of the 24-well plate. The reaction took place in a shaking waterbath at 37°C. The reaction was stopped by transferring the plate on ice and adding 100 μl of 70% perchloric acid. After an incubation time of 1 hour the chromatin papers were collected in scintillation tubes (6.5 ml, Roth #AYX4.1) and measured using a scintillation counter (Tri-Carb 2810TR Low Activity Liquid Scintillation Analyzer, Perkin Elmer). Activity was normalized to tissue weight or cell number respectively as previously described (9). PCR amplified templates (plasmid template: AK079912, Fantom2-cloneID: A530001O10 with appropriate oligonucleotides providing a T7 RNA polymerase promoter (Supplementary Table S1)) for mitolnc and its variants were used to generate RNA to supplement extracts of HEK293 cells or cardiac extracts of mitolnc deficient animals. 5 pmol (cardiac extracts) or 50 pmol (HEK293 extracts) of each *in vitro* transcribed RNA (T7 RiboMaxx™ Express large scale RNA Production System, Promega #P1320) lncRNA variant in combination with 1 μl Digitonin (10 mg/ml) was added to Buffer A. In addition, the activity assay was performed using lysates of HEK293 and HEP2G cells transfected with plasmids (pcDNA3.1) expressing HIS-V5 tagged versions of mouse BCKDH subunits (BCKDHA, BCKDHB, DBT, DLD) with or without a plasmid expressing mitolnc.

### ACLY activity assay

For analysis of ACLY activity, tissue extracts of WT and mitolnc deficient animals were used. Lysates were freshly prepared as described for the IP immediately before starting the assay using modified RIPA (50 mM Tris–HCl pH 7.4, 1% NP-40, 0.25% sodium deoxycholate, 150 mM NaCl, 1× protease inhibitor cocktail III (Merck #539134, 1 U RNasin (Promega #N2611)), 1 x phosSTOP^TM^ (Merck #4906845001). Lysates containing 100 μg of protein were used for the ACLY Assay Kit (Bioscience #79904) according to manufacturer's instructions.

### Sucrose density gradient centrifugation

To analyze the complex stability and density we performed sucrose gradient centrifugation using protein lysates of WT and KO animal hearts as described (43). Protein lysates were prepared as described for IP using modified RIPA (50 mM Tris HCl, 150 mM NaCl: 1% NP40; 0.25% sodium deoxycholate, pH 7.4 with HCl). Protein lysates (1 mg/300 μl) were added on top of a sucrose gradient (5%, 10%, 15%, 20%, 25%, 30%, 35%, 40% sucrose). Samples were centrifuged at 38 300 rpm (250 000 × g; 16 h, 4°C, Beckman coulter optima L30K; rotor: SW41). Thirteen single fractions were collected and proteins were isolated using a final concentration of 7.5% TCA for 4 h at 4°C, followed by 5 min centrifugation at 16 000 × g. Protein pellets were washed twice using 100% acetone followed by 5 min centrifugation at 16 000 × g. Proteins were resuspended in loading buffer and analyzed by western blot. The blots were developed using identical exposure times per target for both genotypes respectively.

### Analysis of coding potential and co-localization

For *in silico* analysis of the coding potential of lncRNAs including mitolnc the tool Coding Potential Calculator 2 (CPC2) was used (http://cpc2.gao-lab.org/). Sequences of mitolnc and previously described lncRNAs Nron, Hotair, the micropeptide encoding RNAs Dworf and Myoregulin as well as the protein-coding RNAs of Acta1 and Gapdh were used. Additionally potential open reading frames were compared to known proteins using the BLAST tool. Fluorescence microscopy images taken from RNA FISH experiments were analyzed for co-localization of signals for mitolnc and atp6 using the coloc2 tool of ImageJ/Fuji with autothreshold. The co-localization coefficient tM1 was used to quantify how many of the pixels detected in channel 2 (mitolnc - red) are co-localized with the detected objects in channel 1 (ATP6 – green). Thereby, 100% of overlap of the signal corresponds to a value of 1 (44).

### Statistical analysis

Statistical analysis was performed using GraphPad Prism V9. Data were tested for normal distribution and Student's *t*-test or the Mann–Whitney test were used accordingly. For multiple comparison the Fisher LSD test was used in the course of a two-way Anova multivariance analysis. Data are represented as mean ± SEM. In case of multiple data points data are represented as Box and Whiskers with the box extending from the 25th to 75th percentiles, the whiskers are drawn from the 10th percentile to the 90th percentile and points below and above are represented by single data points. Statistical significances were reported as *P*-values. For evaluation of proteomics or transcriptomics the data were normalized accordingly. *Z*-scores were calculated for visualization of data.

## Results

### Identification of a cardiac, nuclear-encoded mitochondrial localized lncRNA

**Figure 1.**
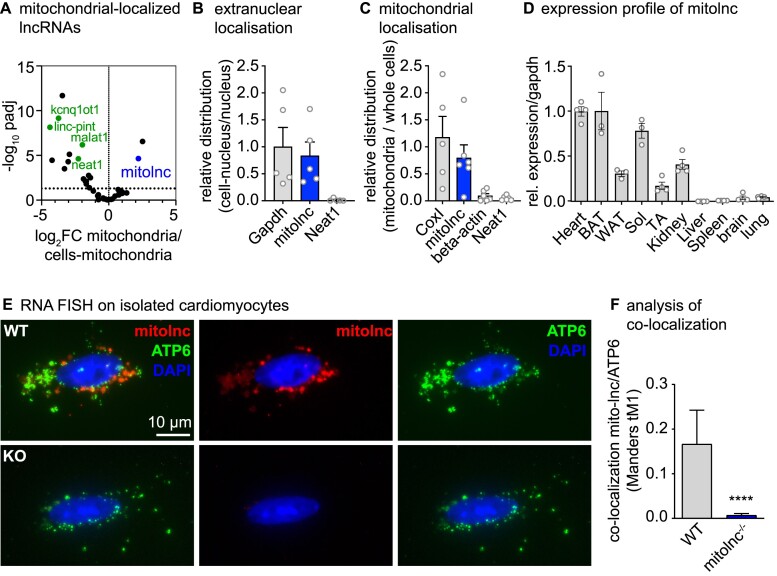
Mitolnc is a nuclear-encoded lincRNA found in mitochondria. (**A**) RNA isolated from mitochondria and of remaining heart lysate analyzed by RNA-Seq. A fold change and an adjusted *P*-value (*P*_adj_) (*n* = 2/2, *P*_adj_< 0.03) were calculated and the result was filtered for Ensembl biotype: lncRNA with a detection of >50 basemean for the heart samples. The non-coding RNA mitolinc (Gm44386, AK079912) was highly enriched in mitochondria. (**B**) RT-qPCR using nuclear and extranuclear fractions of isolated cardiomyocytes. Ratio for (cell-nucleus)/nucleus normalized to the ratio of Gapdh is shown (*n* = 5 animals, extranuclear and nuclear fractions; mean values ± SEM). (**C**) RT-qPCR using mitochondrial fraction and complete heart lysate. Ratio for mitochondria/whole cell normalized to the ratio of Cox1 is shown (Cox1: *n* = 5 animals; mitolnc, Neat1, beta-actin: *n* = 6 animals; mean values ± SEM). (**D**) RT-qPCR for mitolnc in different tissues of the mouse (*n* = 3–4 animals). (**E**, **F**) RNA FISH using probes against mitolnc and Atp6 detect the RNAs outside of the nucleus. The mitolinc signal is lost in mitolnc deficient cardiomyocytes. Co-localization analysis confirms co-localization of mitolnc and Atp6 RNA. Scale bar in d corresponds to 10 μm.

To identify lncRNAs with potential functions in cardiac mitochondria, we extracted RNA from isolated mitochondria and the non-mitochondrial fraction of adult mouse hearts. Comparison of RNA sequencing data for each fraction, followed by filtering for lncRNAs annotated in the Ensembl genome database, identified lncRNAs enriched in mitochondria. The lncRNA GM44386 (AK079912), subsequently named mitolnc, was selected for further analysis (Figure 1A, Supplementary Figure S1A), since nothing is known about its function in the heart, although expression of mitolnc in brown adipose tissue and a role in adipogenesis has been reported before (45,46). Fractionation of adult mouse cardiomyocytes in combination with RT-qPCR demonstrated the localization of mitolnc outside the nuclei (Figure 1B) and RT-qPCR using additional fractionation of heart tissue corroborated localization inside the mitochondrial fraction (Figure 1C). Mitolnc is encoded at mouse chromosome 6qD1 and predominantly expressed in the adult heart, brown adipose tissue (BAT), oxidative skeletal muscle, and kidney. Much weaker or virtually no expression of mitolnc was seen in the liver, brain, lung and spleen (Figure 1D, Supplementary Figure S1B). Mitolnc is detected at lower levels in embryonic compared to adult hearts (Supplementary Figure S1C), which corresponds to the lower numbers of mitochondria in immature cardiomyocytes. Bioinformatic analysis confirmed mitolnc to be ncRNA without any conserved ORF (Supplementary Figure S1D).

To investigate the molecular and physiological functions of mitolnc, we generated two different mitolnc-deficient mouse models either by deleting the mitolnc locus or by inserting a polyA cassette. Deletion of the complete mitolnc locus, resulting in a complete null mutation, was achieved using a CAS9-nickase approach, which reduces potential off-target effects of CAS9 (30). Insertion of the polyA cassette in the first exon by CAS9 also inactivated the mitolnc gene, eliminating all detectable mitolnc expression without affecting expression of the neighboring PCBP1 gene (Supplementary Figure S2A–H). Both mouse lines were viable showing a Mendelian distribution of offspring, without any obvious developmental abnormalities, including normal adipogenesis (Supplementary Figure S2I–M). RNA fluorescent *in situ* hybridization using adult mouse cardiomyocytes and probes directed against mitolnc and the mitochondrial encoded ATP6 corroborated the presence of mitolnc in cardiac mitochondria. No mitolnc signals were detected in mitolnc-deficient cardiomyocytes, demonstrating specificity of the probes (Figure 1E, F).

### Deletion of mitolnc expression induces cardiac hypertrophy in adult animals

**Figure 2.**
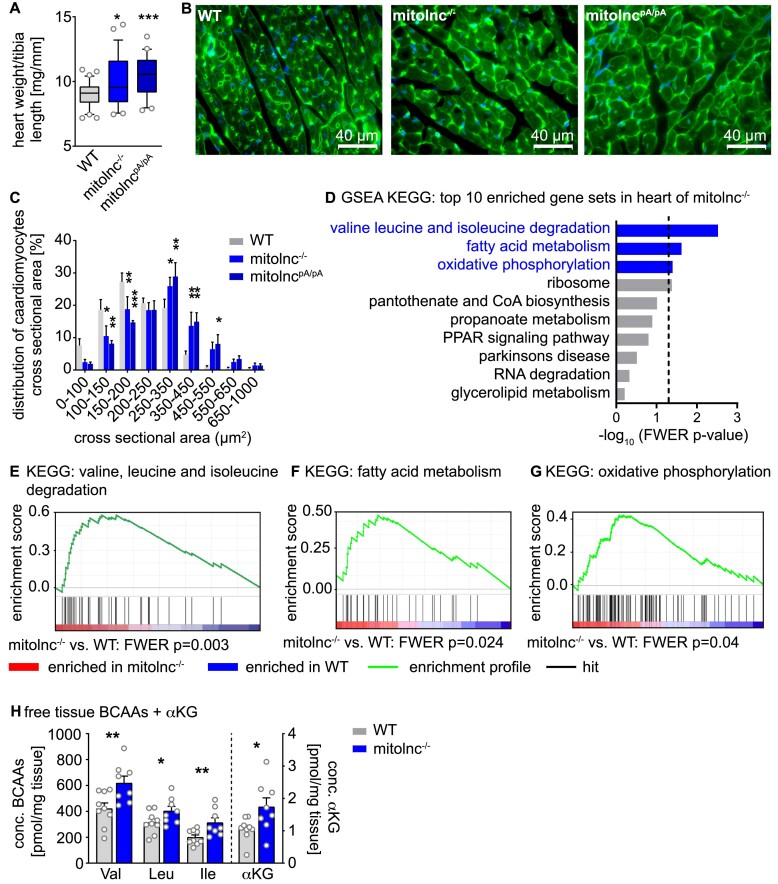
Mitolnc prevents cardiac hypertrophy and controls BCAA catabolism. (**A**) Heart weight of WT, mitolnc^−/−^ and mitolnc^pA/pA^ animals aged 17 ± 1 weeks (box and whiskers, box: 25–75 percentiles, whiskers: 10–90 percentile; *n* = 33 WT, *n* = 26 mitolnc^−/−^, *n* = 20 mitolnc^pA/pA^ animals, unpaired two-tailed *t*-test * *P*< 0.05, *** *P*< 0.001). (**B**) Cross sections of cardiac tissue stained using WGA-Alexa488 to evaluate the cross sectional area of cardiomyocytes. (**C**) Individual cardiomyocytes were grouped according to their cross sectional area and distribution of cross sectional area was analyzed (*n* = 9 WT, *n* = 8 mitolnc^−/−^, *n* = 3 mitolnc^pA/pA^; two-way ANOVA, Fishers LSD test). (**D**–**G**) Transcriptome analysis of WT and mitolnc deficient hearts at 8 weeks of age in combination with gene set enrichment analysis (GSEA) revealed significant enrichment of gene sets associated with mitochondrial functions (*n* = 7 WT, *n* = 8 KO [Del + pA]). (**H**) BCAA levels in heart tissue (*n* = 9 WT, *n* = 8 mitolnc^−/−^ heart samples, Student's *t*-test, one-tailed. Valine: ** *P* = 0.006, Leucine: *P = 0.033, Isoleucine: **P = 0.006, αKG: * *P* = 0.016).

Although inactivation of mitolnc does not cause obvious developmental defects in either mouse model and heart weights were normal at 8 weeks of age (Supplementary Figure S2K), we noted increased heart weights in both mitolnc deficient mouse models at 16 weeks of age (Figure 2A, Supplementary Figure S2L, M). In line with this finding, histological analysis of cardiac tissue at 16 weeks of age revealed an increase of the cross-sectional area of cardiomyocytes (Figure 2B, C). Unbiased examination of transcriptome data by gene set enrichment analysis (39) using KEGG gene sets revealed enrichment of gene sets specific for BCAA catabolism, fatty acid metabolism, and oxidative phosphorylation (Figure 2D). Mitolnc-deficient hearts showed increased gene expression of all three gene sets (Figure 2E–G). Next, we investigated the cardiac metabolome to learn whether the transcriptional changes in mitolnc-deficient hearts are also associated with metabolic changes. Importantly, we observed substantially increased concentrations of all three branched chain amino acids and of α-ketoglutarate, which is probably caused via allosteric activation of GDH by leucine (Figure 2H).

**Figure 3.**
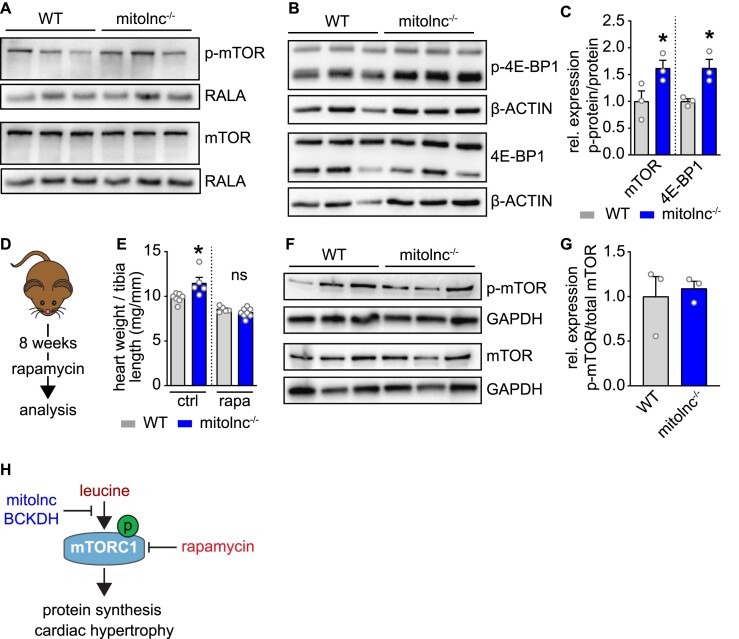
Increased BCAA activates mTOR signaling in the heart. (**A**) Western blot analysis of heart lysates for *p*-mTOR^ser2448^/mTOR (*n* = 3 WT, *n* = 3 mitolnc^−/−^); (**B**) Western blot analysis of heart lysates for *p*-4EB-P1^thr37/46^/4EB-P1 (*n* = 3 WT, *n* = 3 mitolnc^−/−^); (**C**) Evaluation of western blots (one-tailed Mann–Whitney test, * *P* = 0.05). (**D**) Rapamycin diet treatment for 8 weeks in adult WT and mitolnc^−/−^ mice. (**E**) Heart weight/tibia length ratio of WT and mitolnc^−/−^ mice treated with control diet or rapamycin containing diet (control diet: *n* = 7 WT, *n* = 5 mitolnc KO; rapamycin diet: *n* = 5 WT, *n* = 8 mitolnc KO; two-tailed unpaired *t*-tests, * *P* = 0.015, ns = 0.166). (**F**, **G**) Western blot analysis of heart lysates of rapamycin diet treated mice for p-mTOR^ser2448^/mTOR; evaluation of western blot data (*n* = 3 WT, *n* = 3 mitolnc^−/−^; one-tailed *t*-test unpaired; *P* = 0.3623). (**H**) Increased concentration of the branched chain amino acid leucine results in mTOR activation and in cardiac hypertrophy. *In vivo* the mitolnc–BCKDH interaction results in increased degradation of BCAAs and thus prevents hypertrophy. Cardiac hypertrophy due to decreased BCKDH activity after loss of *mitolnc* is rescued by inhibiton of mTOR using rapamycin.

Increased concentrations of leucine are known to activate mTOR signaling, the latter being a well-characterized pathway in the induction of cardiac hypertrophy (1,15,47). To confirm activation of mTOR signaling in mitolnc deficient hearts as a consequence of elevated leucine levels, we performed a western blot analysis, which uncovered increased p-mTOR (Ser2448) and *p*-4E-BP1 (Thr37/46) concentrations in mitolnc-deficient compared to WT hearts (Figure 3A–C). To corroborate the causal relation between increased BCAA levels and activation of mTOR signaling, WT mice were fed with a BCAA-rich chow, which increased BCAA serum levels (Supplementary Figure S3A, B), and also increased phosphorylation of mTOR at serine2448 in hearts (Supplementary Figure S3C, D). Next, we inhibited mTOR signaling in mitolnc-deficient hearts by administration of rapamycin. Suppression of mTOR signaling abrogated differences in heart weight between WT and mitolnc deficient animals, demonstrating that leucine-dependent mTOR activation is responsible for induction of cardiac hypertrophy in mutant animals, although additional mechanisms contributing to cardiac hypertrophy cannot be ruled out (Figure 3D–H, Supplementary Figure S3E–G).

Mitolnc expression is also detected in BAT and skeletal muscle, where its localization is confined to mitochondria of adipocytes and skeletal muscle myofibers, similar as in cardiomyocytes (Supplementary Figure S4A, B). Transcriptome analysis of BAT revealed an enrichment of gene sets related to BCAA and fatty acid metabolism (Supplementary Figure S4C–E), but no significant changes in BCAA concentrations were detected in this tissue (Supplementary Figure S4F), indicating a lower physiological impact of mitolnc in BAT compared to cardiac tissue. No changes in the concentration of free BCAAs were detected in liver and serum of mitolnc deficient mice (Supplementary Figure S4G–I), excluding systemic effects of increased BCAAs concentration on the heart.

### Mitolnc interacts with essential enzymes of the BCAA catabolism

**Figure 4.**
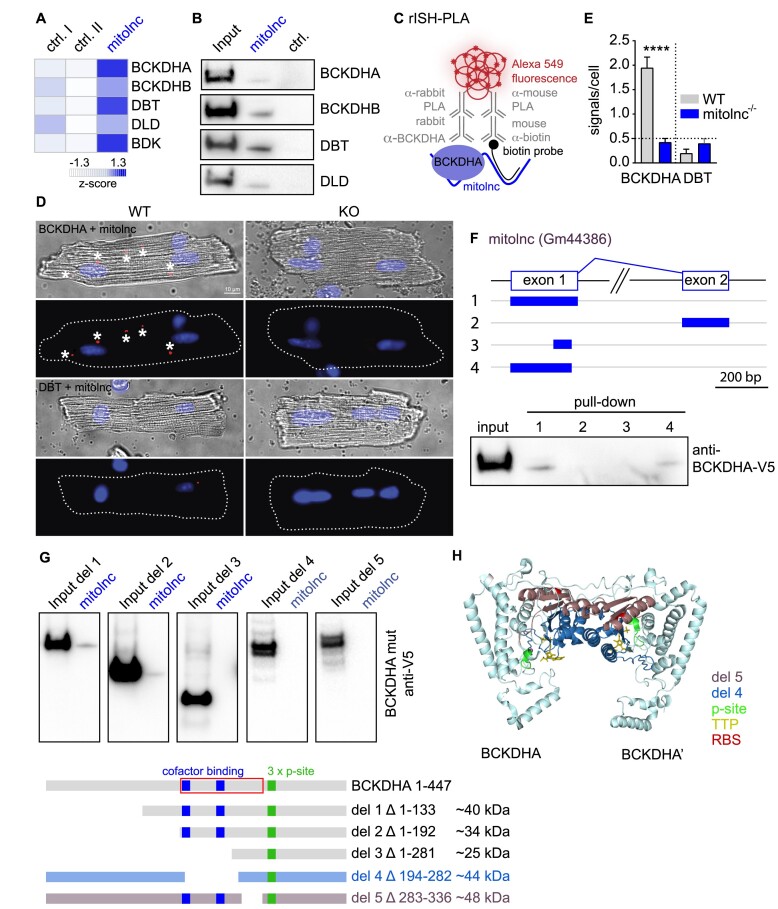
Mitolnc controls the catabolic flux of BCAAs by interacting with BCKDHA. (**A**) RNA–protein pull-down using *in vitro* transcribed mitolnc and protein extracts isolated from heart combined with MS analysis of the bound proteins identifies proteins specifically pulled down by mitolnc (mitolnc + two ctrl-lncRNAs (AK086006; AK080188: lnc-myh), technical triplicates each, *z*-score of averaged detection values). (**B**) The RNA–protein pull down experiment was confirmed by western blot using *in vitro* transcribed RNA in combination with protein extracts of HEK293 cells overexpressing the respective proteins fused to a V5 tag. (**C**) Schematic description of the RNA *in situ* hybridization-proximity ligation assay (rISH-PLA). (**D**) RNA *in situ* hybridisation–proximity ligation assay (rISH-PLA) to detect interaction of mitolnc with BCKDHA or DBT in isolated cardiomyocytes cultured for 12 h. (**E**) Quantification of rISH-PLA (6 wells/condition; BCKDHA: *n* = 55 WT cells, *n* = 83 KO cells; DBT: *n* = 46 WT cells, *n* = 58 KO cells; two-tailed *t*-test ****<0.0001). (**F**) RNA–protein pulldown using *in vitro* transcribed variants of mitolnc and protein extracts of HEK293 cells overexpressing BCKDHA-V5 followed by western blot analysis. (**G**) RNA–protein pulldown using *in vitro* transcribed mitolnc and protein extracts of HEK293 cells overexpressing mutant variants of BCKDHA-V5 followed by western blot analysis. (**H**) Structure of E1α dimer of the BCKDH complex based on PDB#1V1R (doi: 10.2210/pdb1V1R/pdb) with deleted protein domains marked in blue (deletion 4) or brown (deletion 5), TPP at the active site of BCKDHA is marked in yellow, phosphorylation sites (STSDDSSAYRS sequence, 1st serine corresponding to yeast S293 site) are marked in green. Localisation of the direct mitolnc–BCKDHA interaction site identified by UV-crosslinking and HF-MS is marked in red.

To gain insights how the loss of mitolnc increases the concentration of cardiac BCAAs, we searched for proteins potentially interacting with mitolnc by RNA–protein pull-down assays using *in vitro* synthesized lncRNAs coupled to biotin and incubated with cardiac protein lysates. To control for non-specific interactions between RNAs and proteins in this assay, we included two additional control-lncRNAs. RNA interacting proteins were identified by mass-spectrometry, revealing specific interactions of mitolnc with components of the BCKDH complex (Figure 4A). The BCKDH complex mediates the first irreversible step in the catabolism of BCAAs by decarboxylation of BCAA-derived ketoacids. Reduced activity of the BCKDH complex leads to accumulation of BCAAs and causes maple syrup urine disease (MSUD) (3). To confirm the interaction between mitolnc and components of the BCKDH complex, we used mitolnc to pull down V5-tagged BCKDH complex components expressed in HEK293 cells, followed by western blot analysis. We detected interactions of mitolnc with the E1 subunits of the BCKDH complex, comprising BCKDHA and BCKDHB, as well as interactions with the E2/E3 subunits DBT and DLD (Figure 4B).

To corroborate the interaction between mitolnc and the BCKDH complex in an *in vivo* setting, we applied the *RNA in situ hybridisation proximity ligation assay* (rISH-PLA, Figure 4C), which detects molecules in close proximity to each other. rISH-PLA revealed signals outside of the nucleus of isolated adult cardiomyocytes when antibodies against the BCKDHA subunit of E1 together with antibodies against hybridized mitolnc-specific probes were used (Figure 4D, E). In line with the mitochondrial localization of mitolnc and BCKDH, rISH-PLA signals overlapped with MitoTracker Deep Red staining (Supplementary Figure S5A-C). In contrast, no signals were obtained when antibodies against DBT, the E2 component of the BCKDH complex, were employed in this assay, which is in conflict with the results from the pull-down experiments (Figure 4D, E). We reason that the increased concentration of DBT due to overexpression facilitates its indirect pull-down by mitolnc via interaction with BCKDHA, whereas rISH-PLA is done in a physiological setting, avoiding such artefacts and detecting more specifically proximity of the molecules.

For further analysis of the BCKDH–mitolnc interaction, we investigated direct RNA–protein contacts by comparing protein extracts of UV-crosslinked mitochondria isolated from WT and mitolnc^−/−^ hearts followed by hydrofluoric acid treatment and mass spectrometry (HF-MS). After isolation of the cross-linked RNA–protein complexes, phosphodiester bonds of the RNA were cleaved using hydrofluoric acid, which leaves a crosslinked uridine residue of the RNA at peptides (41). Subsequent mass spectrometry analysis identified a uridine-modified, BCKDHA-derived peptide only in WT but not in mitolnc^−/−^ protein extracts (Supplementary Figure S5D–F), further corroborating the interaction of mitolnc with mitochondrial BCKDHA *in vivo*. The analysis also indicated that the interaction of mitolnc with BCKDHA occurs at a site close to essential BCKDHA cofactor binding and regulatory phosphorylation.

To further characterize the interaction of mitolnc and BCKDH complex, we analyzed several mutants of BCKDHA and mitolnc. We found that a part of the first exon of mitolnc, including a sequence motif conserved between mouse and human (Supplementary Figure S1B), is essential and sufficient for the interaction with BCKDHA. In contrast, RNA sequence downstream of the conserved sequence motif in exon 1 and sequences in exon 2 are dispensable for the interaction with BCKDHA (Figure 4F). We found that the first 192 aa of BCKDHA are not essential for the interaction with mitolnc, whereas deletion of the protein domain containing the peptide identified by HF-MS (del5, Figure 4G, H) as well as deletion of the BCKDHA cofactor binding sites abolishes BCKDH-mitolnc interactions.

### Loss of mitolnc reduces the activity of BCKDC and ACLY in cardiomyocytes

**Figure 5.**
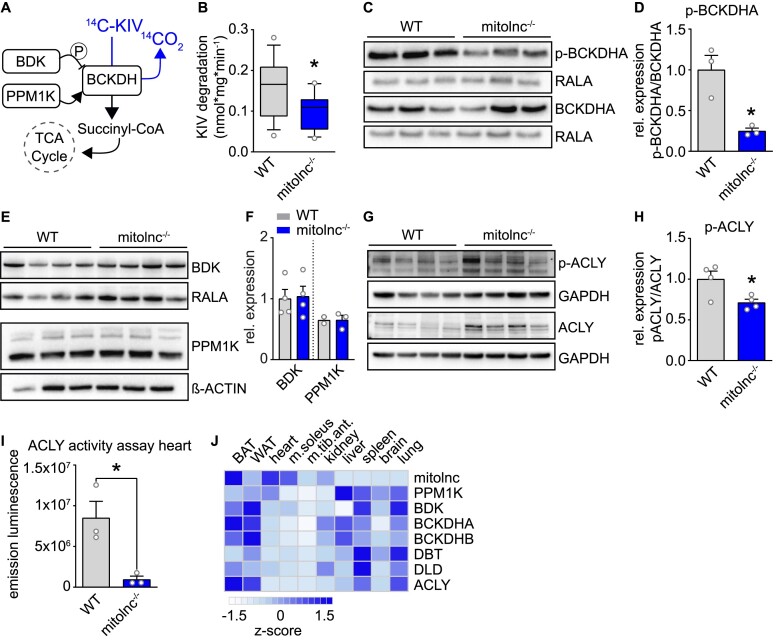
Regulation of BCAA metabolism by mitolnc is independent of phosphorylation. (**A**) Schematic representation of BCKDH complex activity and regulation. (**B**) Catabolism of α-keto-β-methylvaleric acid (KIV; BCKDH activity in nmol/(mg of tissue*min) by mitochondria isolated from hearts of WT and mitolnc KO animals (WT: *n* = 18, *mitolnc* KO: *n* = 18; Student's *t*-test two-tailed, *P* = 0.0135, differences between means ± SEM: –0.053 ± 0.020). (**C**, **D**) Western blot analysis of abundance of BCKDHA and phosphorylation of BCKDHA ser293, (WT: *n* = 3; KO: *n* = 3; Mann–Whitney test one-tailed; *P = 0.05). (**E**, **F**) Western blot analysis of BDK and PPM1K abundance (BDK: WT: *n* = 4; KO: *n* = 4, *t*-test, two-tailed, not significant; PPM1K: WT: *n* = 3; KO: *n* = 3, Mann–Whitney test one-tailed, not significant). (**G**, **H**) Western blot analysis of ACLY abundance and phosphorylation of ACLY ser455 (WT: *n* = 4; KO: *n* = 4; *t*-test, two-tailed; *P = 0.036). (**I**) ACLY activity assay of heart lysates (WT: *n* = 3; KO: *n* = 3; *t*-test, two-tailed; *P = 0.0218) (baseline = recombinant ACLY protein activity). (**J**) Tissue specific expression of genes associated with the BCKDH complex based on Taqman gene expression analysis (*n* = 3–4 animals).

The accumulation of BCAAs in the heart of mitolnc-deficient mice made it likely that mitolnc is required to achieve high enzymatic activity of BCKDH. To directly test this possibility, we determined the activity of the BCKDH complex in WT and mitolnc-deficient heart tissue. We used an assay in which the release of ^14^C from ^14^C labelled ketoacid of valine α-keto [1-^14^C] isovalerate (^14^C KIV) is employed to measure the activity of the BCKDH complex (Figure 5A) (9). We observed a significant reduction of BCKDH complex activity in mitolnc-deficient compared to WT control hearts (Figure 5B), despite equal protein levels of BCKDH complex components (Supplementary Figure S6). Loss of mitolnc might reduce BCKDH complex activity due to various reasons, including changes in the subcellular localization of the BCKDH complex or alteration of posttranslational modifications. First, we tested whether mitolnc affects the transport of BCKDH complex components. Subcellular fractionation, followed by western blot analysis of the different fractions did not reveal any differences in the mitochondrial localization of BCKDH complex components between WT controls and mitolnc-deficient hearts (Supplementary Figure S6). Next, we analyzed changes in the phosphorylation of BCKDH, since its activity is suppressed by phosphorylation at Ser293 of BCKDHA (Figure 5A). Surprisingly, we observed decreased phosphorylation of BCKDHA in mitolnc-deficient hearts (Figure 5C, D), although decreased phosphorylation normally indicates high enzymatic activity of the BCKDH complex.

Phosphorylation of BCKDHA is accomplished by the branched chain ketoacid dehydrogenase kinase (BDK) and counteracted by the protein phosphatase 1K (PPM1K), which dephosphorylates BCKDHA to increase its activity. We found that the reduced phosphorylation of BCKDHA by BDK is not associated with changes in the abundance of BDK or PPM1K, as indicated by western blots analysis (Figure 5E, F), but most likely caused by allosteric inhibition of BDK via α-ketoisocaproate (KIC) derived from increased concentrations of leucine. KIC accumulates upon increase of leucine and represents an important component of the feedback loop, activating BCKDH to cope with an increased load of BCAAs (48,49). The results also underscore the importance of mitolnc for achieving high BCKDH complex activity, since BCKDH complex activity was low in mitolnc-deficient hearts, despite massive dephosphorylation of BCKDHA.

Interestingly, we also detected decreased phosphorylation of the ATP citrate lyase (ACLY) (Figure 5G, H), required for cytosolic generation of acetyl-CoA for *de novo* lipogenesis but also for epigenetic regulation through histone acetylation (50). ACLY was recently described to be co-regulated by the BDK/PPM1K system to allow integration of BCAA and lipid metabolism (9). In line with the lowered phosphorylation of ACLY, we found strongly decreased ACLY activity in cardiac lysates (Figure 5I). We also observed an inverse correlation between expression of mitolnc and PPM1K/BDK/ACLY in some tissues, suggesting that activation of BCAA catabolism by mitolnc primarily happens in tissues, in which expression of components of the BDK/PPM1K/ACLY regulon is comparatively low (Figure 5J). In conclusion, the decreased activity of ACLY in mitolnc-deficient hearts may reflect the requirement of some cell types to regulate BCKDH complex activity independently of BDK and PPM1K, i.e. to stimulate activity of the BCKDH complex independent of BDK, thereby avoiding direct effects on ACLY.

### Mitolnc allosterically activates the BCKDH complex

We found that mitolnc does neither affect mitochondrial localization of the BCKDH complex nor increase repressive phosphorylation of BCKDHA, which leaves a role of mitolnc in the assembly of the BCKDH complex or a direct function as allosteric activator as potential modes of action. To analyze whether loss of mitolnc affects composition of the BCKDH complex, we immunoprecipitated the BCKDH complex from heart lysates of WT and mitolnc-deficient hearts using antibodies directed against DBT and MCCA (Supplementary Figure S7A, B) followed by mass spectrometry. We quantitatively identified all components of the BCKDH complex including BCKDHA, BCKDHB, DBT and DLD in both IP experiments as well as additional proteins annotated to belong to the BCAA degradation pathway, without detecting any apparent differences in complex composition between WT and mitolnc-deficient hearts (Supplementary Figure S7C–O). The unchanged composition of the BCKDH complex in mitolnc-deficient hearts strongly argues against a function of mitolnc as an adaptor to facilitate assembly of the complex or to recruit additional components.

**Figure 6.**
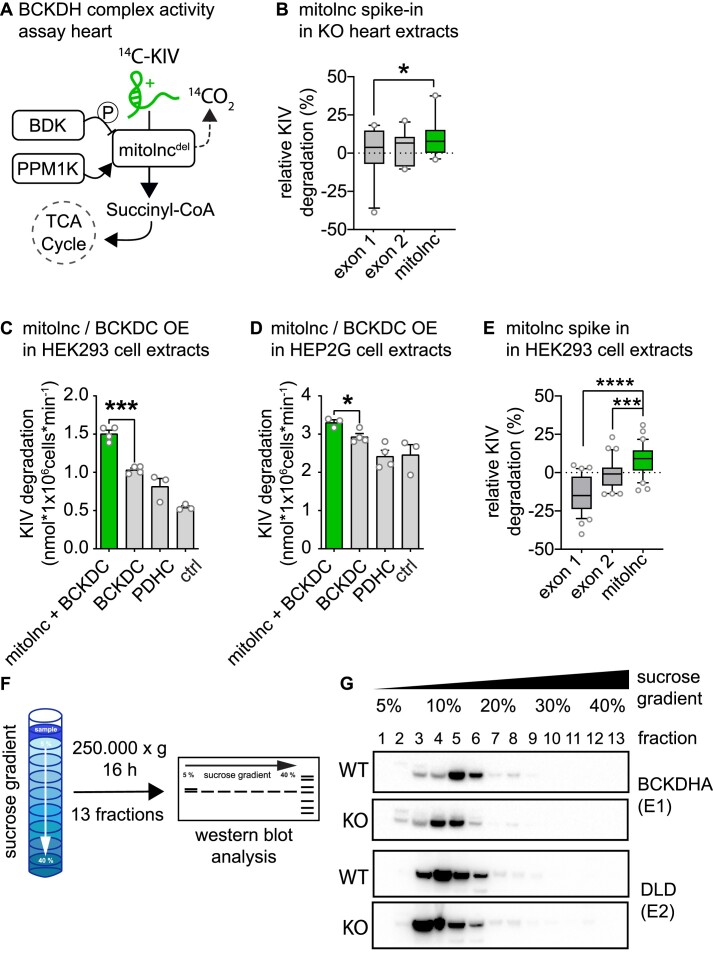
Mitolnc increases metabolization of KIV. (**A**) Schematic representation of BCKDH complex (BCKDC) action and regulation in the experimental setup for BCKDH complex activity analysis. (**B**) Relative degree of KIV metabolization in tissue extracts of mitolnc^−/−^ hearts after addition of *in vitro* synthesized mitolnc or mutant variants (5 pmol). Values were normalized to mitolnc^−/−^ heart extracts (*n* = 10 each variant; one-tailed paired *t*-test, * *P* = 0.0461). (**C**) KIV degradation in HEK293 cells transfected with plasmids expressing all BCKDH complex subunits + plasmid expressing mitolnc, all BCKDH complex subunits, pyruvate dehydrogenase complex subunits or control plasmid (*n* = 4 BCKDH complex + mitolnc; *n* = 4 BCKDH complex; *n* = 3 PDH; *n* = 3 (ctrl = px458); two-tailed *t*-test; ***P = 0.0001). (**D**) KIV degradation in Hep2G cells transfected with plasmids expressing all BCKDH complex subunits + mitolnc expressing plasmid, all BCKDH complex, pyruvate dehydrogenase complex subunits or control plasmid (*n* = 3 BCKDHC + mitolnc; *n* = 4 BCKDC; *n* = 4 PDHC; *n* = 3 p900 (ctrl = px458); two-tailed *t*-test; * *P* = 0.0223). (**E**) Relative KIV degradation in HEK293 cell lysates after addition of *in vitro* synthesized mitolnc or mutant variants of mitolnc (50 pmol). Values were normalized to the mean activity in HEK293 cell lysate (*n* = 32 HEK293 + exon1, *n* = 30 HEK293 + exon2, *n* = 30 HEK293 + mitolnc; two-tailed *t*-test, *** *P* = 0.0006, **** *P* < 0.0001). (**F**) Schematic representation of sucrose gradient centrifugation for heart protein extracts. (**G**) Sucrose gradient centrifugation was performed for protein extracts isolated from hearts of WT and mitolnc^−/−^ (KO) animals; fractions were analyzed for distribution of BCKDHA, DLD and DBT by western blot analysis.

Next, we tested whether mitolnc might directly stimulate BCKDH complex activity by acting as an allosteric activator. We added *in vitro* transcribed mitolnc to cardiac extracts of mitolnc deficient mice and observed a significant increase in BCKDH complex activity, whereas addition of mutant variants of mitolnc did not have any effects (Figure 6A, B). Moreover, we expressed the BCKDH complex either with mitolnc or its mutant variants in HEK293 and Hep2G cells. Inclusion of full length mitolnc increased activity of the BCKDH complex in HEK293 and Hep2G cells, which normally do not express mitolnc (Figure 6C, D). We also detected increased BCKDH complex activity when adding *in vitro* transcribed mitolnc to extracts of HEK293 cells (Figure 6E), conclusively demonstrating that mitolnc directly enhances the enzymatic activity of the BCKDH complex. Since HEK293 are of human origin, these results also indicate that the mechanism of lncRNA-dependent enzyme activation is conserved between species. To corroborate the hypothesis that mitolnc acts by direct allosteric activation of BCKDH, probably by changing conformation of the complex, we analyzed fractions of sucrose density gradients loaded with extracts from WT and mitolnc deficient hearts. The BCKDHA and DLD subunits of the BCKDH complex were detected in the same fractions of the gradient when comparing WT and mitolnc-deficient hearts. However, absence of mitolnc shifted the localization of BCKDHA and DLD subunits to a different fraction with lower density (Figure 6F, G), suggesting changes in protein conformation.

## Discussion

Here, we describe the unexpected function of the long non-coding RNA mitolnc as an allosteric activator of the mitochondrial BCKDH complex and its role in regulating cardiac metabolism. We found that allosteric activation of the BCKDH complex by mitolnc is critical to reduce local concentrations of leucine in cardiomyocytes, thereby preventing unwanted mTOR activation and cardiac hypertrophy. Our work discloses a previously unknown mechanism of BCKDH complex regulation that allows control of BCAA catabolism independent of BDK/PPM1K-mediated control of phosphorylation of BCKDHA, essentially decoupling BCCA catabolism from generation of citrate-derived acetyl-CoA by ACLY.

BDK/PPM1K-dependent phosphorylation of BCKDHA is high in the heart, suggesting inhibition of BCKDH complex enzyme activity, despite the high oxidative flux of BCAA (51), which is difficult to understand. Availability of substrates, concentration of reaction products as well as allosteric regulation of BCAA decarboxylation has been discussed to explain this obvious contradiction, but no convincing explanation was provided. Here, we offer a solution for this conundrum by identifying mitolnc as an allosteric activator of the BCKDH complex, independent of its phosphorylation status. Mitolnc expression is highest in the heart, exactly the organ in which an allosteric activator is needed to counteract BDK-mediated phosphorylation, resulting in suppression of BCKDH complex activity.

Interestingly, BDK and the corresponding phosphatase PPM1K also control activity of ACLY and potentially other metabolic enzymes (9). ACLY is part of the citrate-malate shuttle, transferring acetyl-CoA from mitochondria to the cytoplasm. In contrast to BCKDHA, phosphorylation of ACLY by BDK-mediated phosphorylation increases ACLY activity, coupling BCAA metabolism with cytosolic generation of acetyl-CoA and fatty acid synthesis. Under homeostatic conditions *de novo* lipogenesis does not play an important role in heart physiology but becomes a critical limiting factor during the stress of acute pressure overload and aging (52). Moreover, ACLY plays a pivotal role for supplying acetyl-CoA, required for epigenetic regulation of cardiomyocytes through histone acetylation (50), which is particularly important for the control of cardiac growth during development and for stress-dependent cardiac remodelling (53). The regulation of BCKDH complex activity by mitolnc avoids the cross-talk to ACLY by uncoupling control of enzyme activity from phosphorylation, thus promoting a high activity of the BCKDH complex to remove high concentrations of BCAAs, even under conditions when a high activity of ACLY is required. The importance to keep BCAA concentrations in cardiomyocytes low becomes apparent from the development of cardiac hypertrophy in mitolnc-deficient mice but also after feeding large amounts of BCAAs.

Apparently, the requirement for uncoupling BCKDHA phosphorylation from ACLY activity differs between different organs, which is reflected by the tissue-specific expression of mitolnc. Our work indicates that organs have to cope with increased BCAA loads locally by adjusting catabolic pathways. We found that reduction of the BCKDH complex activity in mitolnc-deficient mice resulted in a local increase of BCAA concentrations in the heart, without an increase in the serum of mitolnc-deficient mice, which seems to exclude export of BCAAs from cardiomyocytes as a means to lower BCAA concentrations in the heart. We also noted an inverse correlation of the two molecules that activate the BCKDH complex by different mechanisms. Mitolnc is highly expressed in heart, brown adipose tissue, oxidative skeletal muscle, and kidney, whereas expression of PPM1K is lower in these tissues but much stronger in the liver, spleen, lung and brain, indicating organ-specific requirements. So far, we did not identify regulatory stimuli that change expression of mitolnc. Further work is needed to understand whether mitolnc contributes to the dynamic adjustment of BCKDH activity under different disease or metabolic conditions. In contrast to the tissue-specific activation of the BCKDH complex evoked by mitolnc, the regulation of BCKDHA phosphorylation by BDK followed an established pattern. The increase of BCAAs in mitolnc-deficient hearts most likely reduces phosphorylation of BCKDHA at Ser293 (4,54,55), to increase enzyme activity. The reduced phosphorylation of BCKDHA is clearly part of a compensatory cellular response to increase enzyme activity caused by accumulation of BCAAs. Concentrations of BCAAs were in equilibrium with the corresponding branched chain ketoacids (BCKAs) due to the reversible BCAT-mediated transamination, which explains the increase of BCAAs, although BCKAs and not BCAAs are the direct substrates of the BCKDH complex.

The identification of mitolnc as an allosteric activator of the mitochondrial BCKDH complex was a surprise but not completely unexpected. LncRNAs serve multiple different roles ranging from functions in chromatin and transcriptional control to the regulation of protein complex assembly. Many lncRNAs are retained in the nucleus but some are transported to different subcellular structures, suggesting specific functions at these sites (24). Localization of nuclear encoded lncRNA in mitochondria is not common and we are not aware of another published lncRNA that is located in cardiac mitochondria, although some examples of mitochondrially localized lncRNAs exist for other cell types (25,26). The mechanisms enabling transfer of lncRNAs are not understood very well but described to be supported by the polynucleotide phosphorylase (PNPASE) located at the mitochondrial intermembrane space (56). RNA sequence elements essential for the import of RNA into mitochondria have been identified (56), but no consensus sequence motif is known that is necessary and sufficient for RNA import into mitochondria. The abundance of some mitochondrial localized lncRNAs depends on the RNA binding protein GRSF1, located in the mitochondrial matrix (25). Our data demonstrate localization of mitolnc within mitochondria and establish functional interaction with the BCKDH complex in the mitochondrial matrix. At present, we do not know how mitolnc enters mitochondria, i.e. whether the import happens concomitant with the transport of BCKDH complex components, independently via known mitochondrial transporters, or by novel pathways. In the heart of mitolnc deficient animals we observe cardiac hypertrophy and increase of BCCAs. Attenuation of BCAA-induced mTOR activity by treatment with rapamycin prevented cardiac hypertrophy and restored a normal phenotype in mitolnc-deficient animals. These data strongly suggest -together with the localization of mitolnc in mitochondria- that the role of mitolnc is restricted to the regulation of BCKDH, although we do not want to exclude potential other functions.

Our results provide striking evidence that mitolnc directly stimulates BCKDH activity by an allosteric mechanism. We excluded indirect effects, such as an impact on the mitochondrial localization of BCKDH complex components and changes in the composition of the complex. Addition of mitolnc to extracts isolated from mitolnc deficient hearts or HEK293 cells increased the activity of the respective BCKDH complex, which in our view is a strong argument for allosteric activation. It might still be argued that mitolnc recruits or repels an unknown protein not detected in our IP experiments, which alters BCKDH complex activity, although we consider such a scenario unlikely. Moreover, we found that the presence of mitolnc changes migration of the BCKDH complex in sucrose gradients, probably by changing conformation and density of complex components. Finally, analysis of mutant BCKDHA subunits indicates that mitolnc closely interacts with the active center of the BCKDH complex core subunit BCKDHA. Future structural studies of the BCKDH complex in presence or absence of mitolnc using cryoEM or similar techniques might reveal more exhaustive insights into the molecular mechanism of the mitolnc-BCKDH interaction.

Expression and function of mitolnc were analyzed in *Mus musculus*. The human genome contains syntenic sequence elements conserved between mouse and human and this conserved sequence corresponds to the first exon of mouse mitolnc that is important for the interaction with BCKDHA. However, we have not been able to detect mitolnc RNA expression in humans, which might be due to technical limitations. Importantly, we demonstrated that mitolnc increases activity of the BCKDH complex in extracts of human HEK293 cells, clearly indicating that the regulatory principle exploited by mouse mitolnc is active in humans too. It also seems possible that the transcript of mouse mitolnc was not conserved in humans but was replaced by another lncRNA with a different primary sequence but similar conformation, still allowing allosteric activation of human BCKDH complex. Even if a human homologue of mitolnc does not exist, because of different physiological needs and divergent evolution of mammals, the ability to change enzymatic activity of human BCKDH complex by lncRNAs is clearly conserved. Thus, it will be possible to use lncRNAs for manipulation of aberrant BCAA metabolism in humans, which contributes to heart failure and other cardiac diseases in numerous contexts (1).

## Supplementary Material

gkae226_Supplemental_Files

## Data Availability

RNA-Seq data were submitted to NCBI GEO (www.ncbi.nlm.nih.gov/geo #GSE235581, GSE235759 and GSE235762. Microarray data were submitted to the ArrayExpress (http://www.ebi.ac.uk/biostudies/ #E-MTAB- 13047, #E-MTAB-13048. Mass spectrometry data were submitted to ProteomeXchange (RNA–protein pulldown: #PXD044737; IP: PXD044819; HF-MS: PXD049691).
