# Supplementary material for: Mitolnc controls cardiac BCAA metabolism and heart hypertrophy by allosteric activation of BCKDH
Source: Nucleic Acids Res. 2024 Apr 3;52(11):6629–46. doi: 10.1093/nar/gkae226 (PMC11194096; doi:10.1093/nar/gkae226)
Supplement: gkae226_Supplemental_Files [file gkae226_supplemental_files.zip › Weiss et al. Supplementary Information_R2.pdf]

Supplementary information

for

Mitolnc controls cardiac BCAA metabolism and heart hypertrophy by allosteric activation of BCKDH.

Maria Weiss, Sara Hettrich, Theresa Hofmann, Salma Hachim, Stefan Günther, Thomas Braun*, Thomas Boettger*

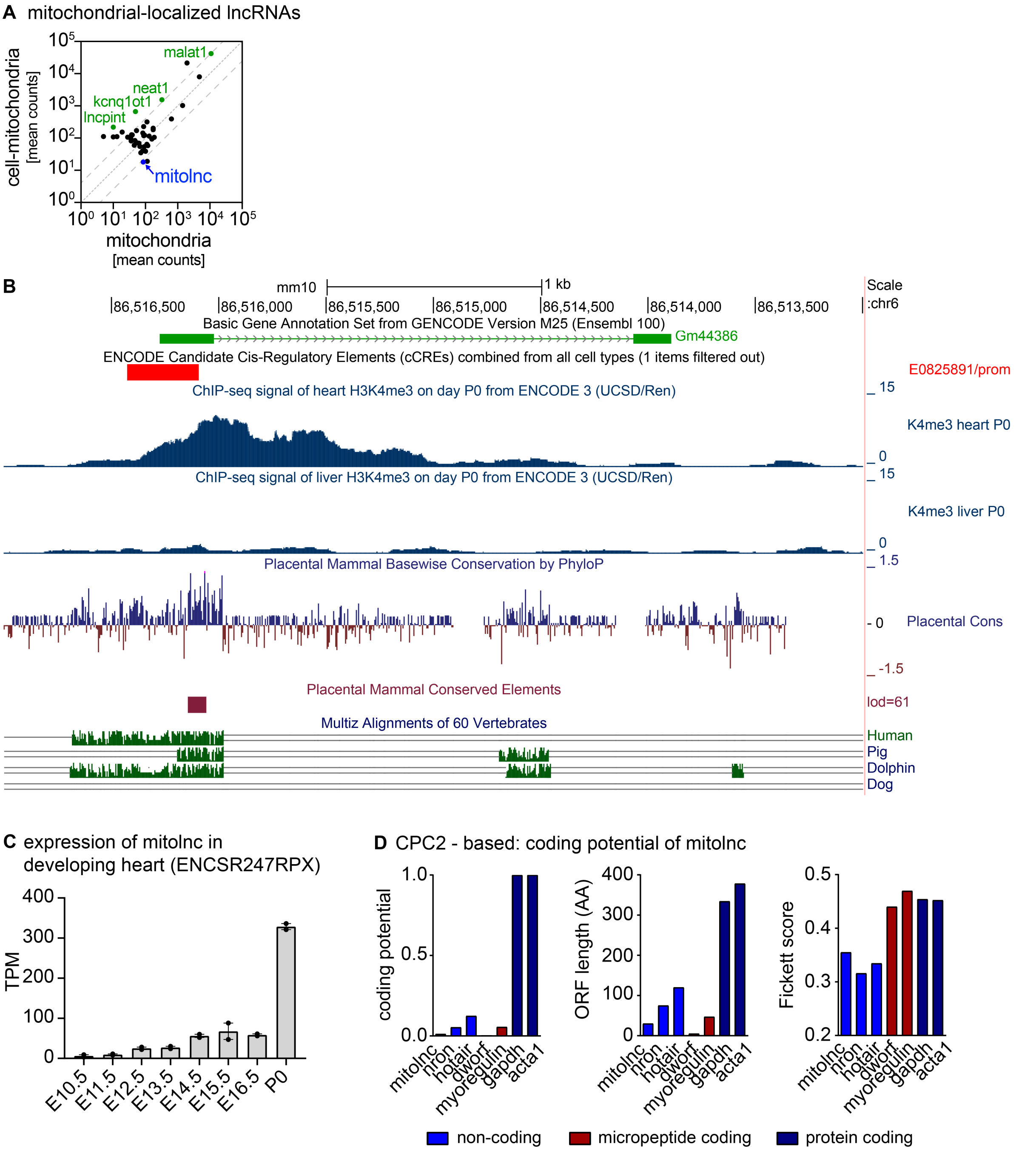

Supplementary Figure S1: Mitolnc is a mitochondrially localized lincRNA, encoded on mouse chromosome 6qD1.

(**A**) Scatter plot of RNA-Seq data using the normalized mean counts for the mitochondrial and non-mitochondrial (rest) fractions of the heart. Log_{10} scales are used, dashed lines indicate a 4-fold change (log_2 = 2). Malat1, Neat1 are highly abundant and enriched in the rest-fraction; Kcnq1ot1 and lncpint as less abundant and highly enriched in the rest-fraction. lncpint is barely detectable in the mitochondrial fraction. Mitolnc is a less abundant lncRNA robustly detected in the mitochondrial fraction (approx. 100 counts), but essentially absent in the rest-fraction.

(B) mitolnc (GM44386, GENCODE M25) is encoded on Mus musculus chromosome 6qD1 (GRCm38/mm10). Data are visualized using the UCSC genome browser using ENCODE and conservation tracks. Encode Candidate Cis-Regulatory Elements (cCREs) indicate a promoter at the 5' region of mitolnc (DOI: 10.1038/s41586-020-2493-4). H3K4me3 ChIP-Seq data (DOI: 10.1093/nar/gkz945) indicate chromatin activity in heart, but not in liver. Conservation data were visualized using the UCSC Genome browser tracks (Vertebrate Multiz Alignment & Conservation) using the Placental Mammal Basewise Conservation by PhyloP, the Placental Mammal Conserved Elements (score > 400) and the Multiz Alignments of 60 Vertebrates sub-tracks. The conservation data indicate conserved sequences for the first exon of mitolnc, while sequences of the second exon are not conserved based on current algorithms.

(C) Expression of mitolnc during cardiac development based on ENCODE RNA seq data ENCSR247RPX.

(D) Coding potential calculator 2 (CPC2) analysis for known non-coding, micropeptide-coding and protein-coding RNAs (http://cpc2.gao-lab.org/). Coding potential, length of potential ORFs and the Fickett score are presented. While coding potential and length of ORF reveal protein-coding RNAs, the Fickett score distinguishes micropeptide-coding RNAs from non-coding RNAs.

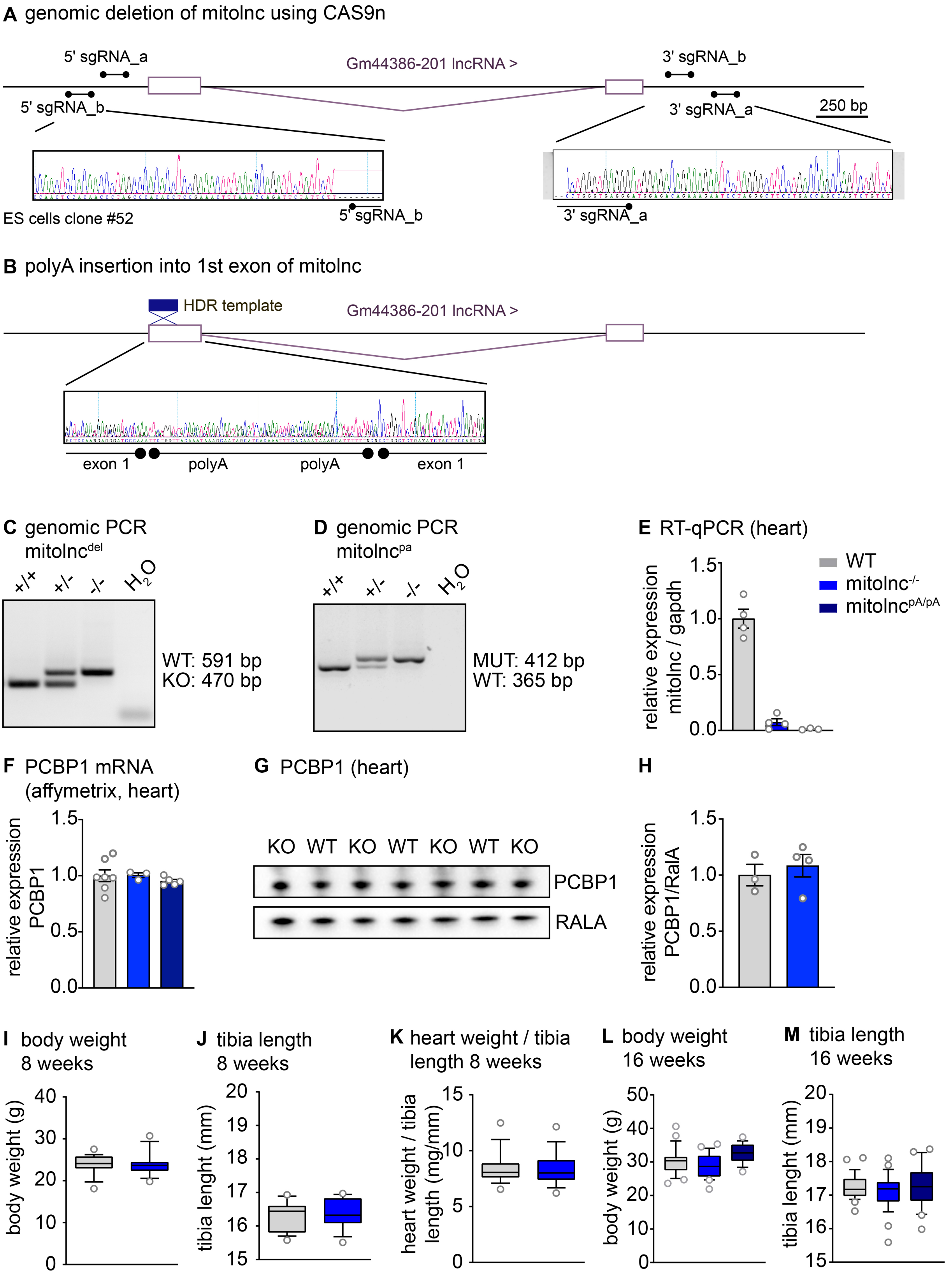

Supplementary Figure S2: Loss of function models for mitolnc.

(A) Deletion of the genomic sequence coding for mitolnc (GM44386) at mouse chromosome 6 using a CAS9n double nickase approach. Two sgRNAs directed against genomic sequence 5' of GM44386 and

two sgRNA directed against genomic sequence 3' of GM44386 in combination with CAS9n were used to induce two pairs of nicks resulting in the genomic deletion of GM44386 in mouse embryonic stem cells.

(B) Homology directed repair was used in combination with an sgRNA / CAS9 to insert a double polyA sequence into the 5' region of the 1st exon of GM44386 in mouse embryonic stem cells.

(C) Mice were generated from embryonic stem cells after deletion of GM44386 and heterozygous mice were bred to homozygosity. PCR using genomic DNA isolated from mice indicates the presence of the modified GM44386 locus.

(D) Mice were generated from embryonic stem cells after insertion of polyA sequence into GM44386 and heterozygous mice were bred to homozygosity. PCR using genomic DNA isolated from mice indicates the presence of the polyA-modified GM44386 locus.

(E) RT-qPCR confirms the absence of the mitolnc transcript encoded by the GM44386 locus in RNA isolated from hearts of homozygous mitolnc$^{-/-}$ (deletion; n = 4) and homozygous mitolnc$^{pA/pA}$ (polyA insertion, n = 3) mice compared to WT mice (n = 4).

(F) Relative expression of PCBP1 mRNA in mitolnc$^{-/-}$ and mitolnc$^{pA/pA}$ compared to WT mouse hearts by Affymetrix gene expression analysis (probe-set: TC0600002639.mm.1; n = 7 WT, n = 3 mitolnc$^{-/-}$, n = 5 mitolnc$^{pA/pA}$; two-tailed t-tests, WT vs. mitolnc$^{-/-}$: ns, p = 0.954, WT vs. mitolnc$^{pA/pA}$: ns, p = 0.460).

(G, H) Western blot analysis of PCBP1 relative to RALA in heart samples (n = 3 WT, n = 4 mitolnc$^{-/-}$; two-tailed t-test: ns, p = 0.585)

(I) Body weight of 8 weeks old mitolnc$^{-/-}$ mice compared to WT mice (n = 17 WT, n = 14 mitolnc$^{-/-}$; two-tailed t-test, ns: p = 0.993).

(J) Tibia length of 8 weeks old mitolnc$^{-/-}$ mice compared to WT mice (n = 17 WT, n = 14 mitolnc$^{-/-}$; two-tailed t-test, ns: p = 0.611).

(K) Heart weight of 8 weeks old mitolnc$^{-/-}$ mice compared to WT mice (n = 17 WT, n = 14 mitolnc$^{-/-}$; two-tailed t-test, ns: p = 0.787).

(L) Body weight of 16 weeks old mitolnc$^{-/-}$ and mitolnc$^{pA/pA}$ mice compared to WT mice (n = 26 WT, n = 22 mitolnc$^{-/-}$, n = 16 mitolnc$^{pA/pA}$; two-tailed t-test WT-del: ns; p = 0.22; WT-pA: p = 0.031).

(M) Tibia length of 16 weeks old mitolnc$^{-/-}$ (n = 27) and mitolnc$^{pA/pA}$ (n = 20) mice compared to WT mice (n = 29 WT, n = 26 mitolnc$^{-/-}$, n = 20 mitolnc$^{pA/pA}$; two-tailed t-test wt-del: ns; p = 0.231; wt-pA: ns; p = 0.870).

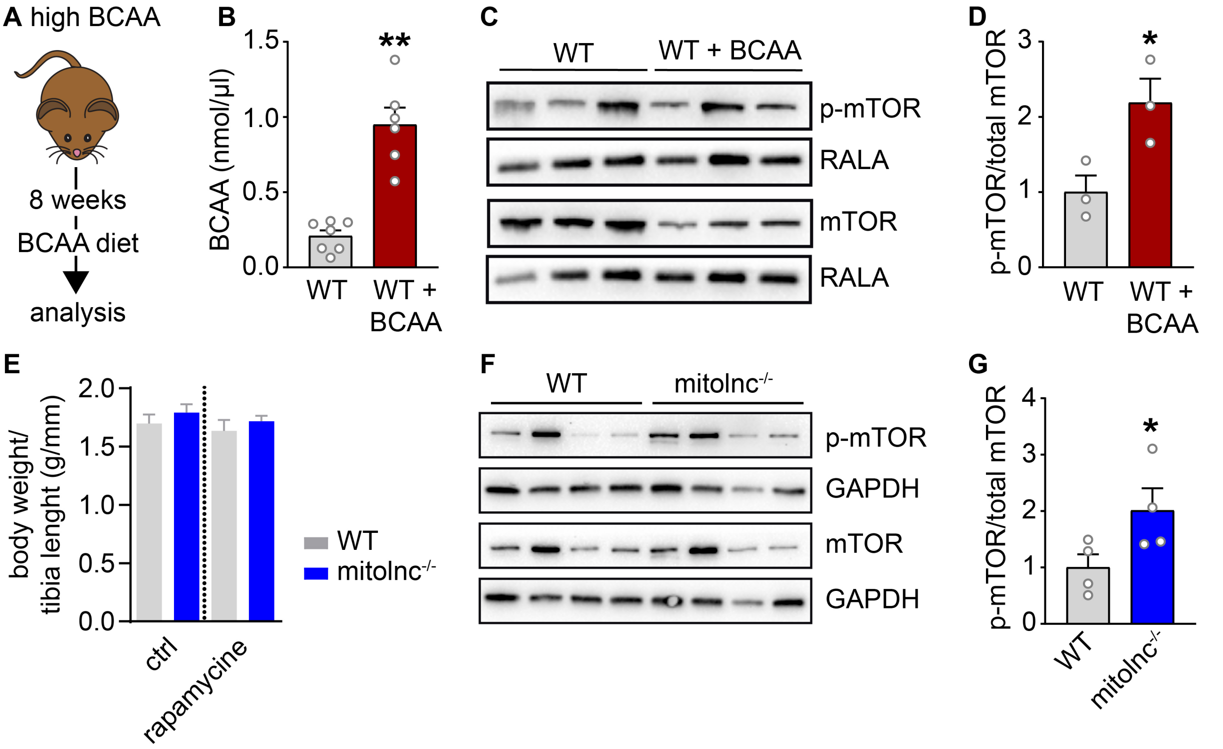

Supplementary Figure S3: Feeding of BCAAs activates mTOR signaling in the heart.

(A) High BCAA diet for 8 weeks in adult mice.

(B) Serum levels of BCAAs after BCAA diet (n = 7 WT, n = 6 WT BCAA diet; two-tailed Mann-Whitney test, ** p = 0.0012).

(C, D) Western blot of heart lysates for WT mice after normal or high BCAA diet for p-mTOR^{ser2448} and mTOR; (D) evaluation of western blot data (n = 3 WT, n = 3 WT + BCAA diet; one-tailed Mann-Whitney test, * p = 0.05)

(E) Body weight / tibia length ratio of WT and mitolnc$^{-/-}$ mice treated with control diet or rapamycin containing diet (control diet: n = 7 WT, n = 5 mitolnc KO; rapamycin diet: n = 5 WT, n = 8 mitolnc KO).

(F, G) Western blot analysis for WT and mitolnc$^{-/-}$ mice fed with control diet fed mice: p-mTOR^{ser2448} / mTOR. (G) evaluation of western blot data (n = 4 WT / 4 KO samples; one-tailed t-test; p = 0.0347).

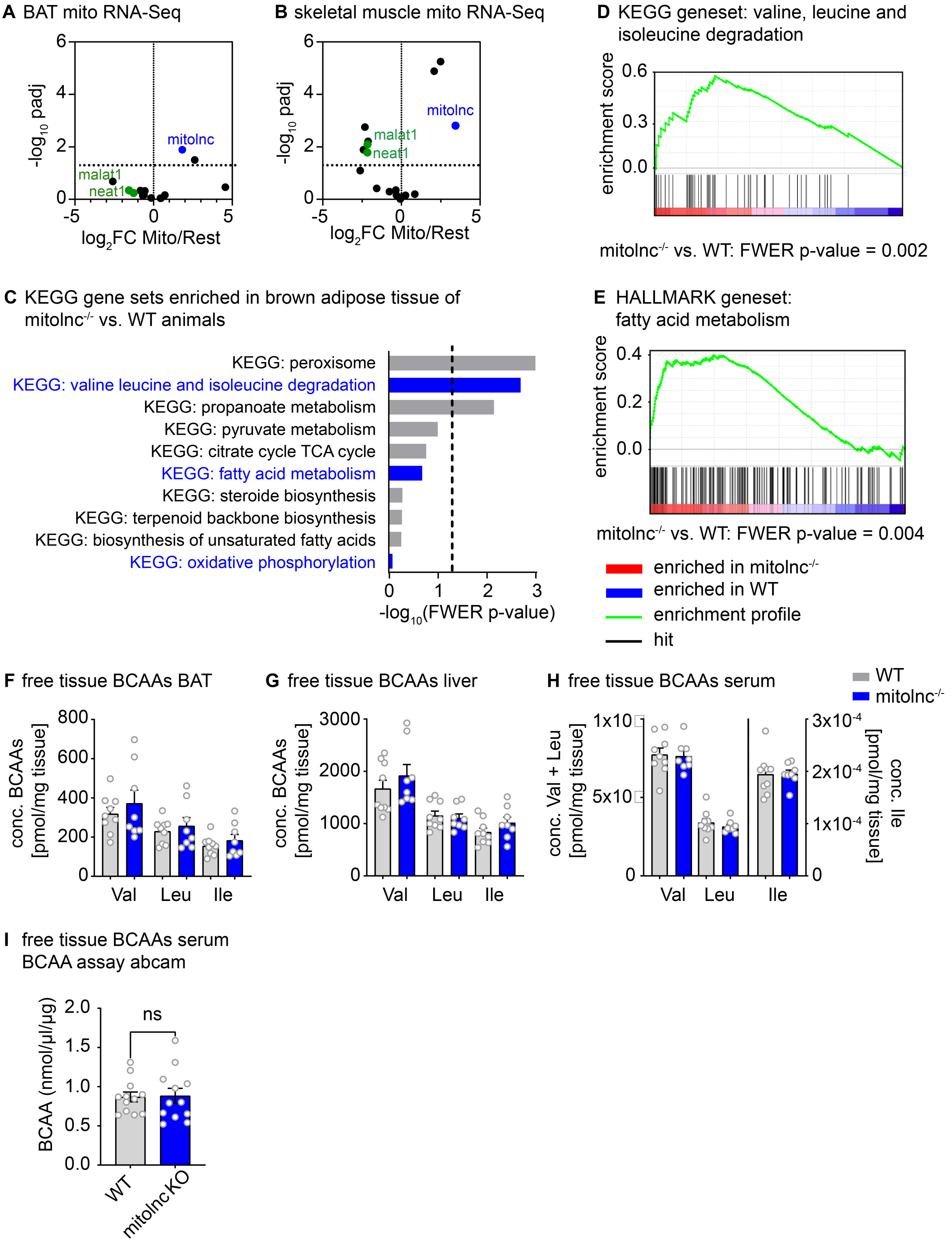

Supplementary Figure S4: Lower impact of mitolnc in brown adipose tissue (BAT) and skeletal muscle.

(A) RNA isolated from mitochondria and from total BAT lysates analyzed by RNA-Seq. A fold change and an adjusted p-value (padj) (n = 2/2) were calculated and the result was filtered for Ensembl

biotype: lncRNA with a detection of > 20 basemean. The non-coding RNA mitolnc (Gm44386, AK079912) was highly enriched in mitochondria.

(B) RNA isolated from mitochondria and from total skeletal muscle lysates were analyzed by RNA-Seq. A fold change and an adjusted p-value (padj) (n = 2/2) was calculated and the result was filtered for Ensembl biotype: lncRNA with a detection of > 20 basemean. The non-coding RNA mitolnc (Gm44386, AK079912) was highly enriched in mitochondria.

(C) Gene set enrichment analysis of transcriptome data for brown adipose tissue isolated from WT and mitolnc$^{-/-}$ animals (n = 4/4) revealed significant enrichment (FWER p-value) of gene sets including BCAA degradation.

(D) Enrichment plot for the KEGG geneset collection: BCAA degradation using brown adipose tissue transcriptome data of mitolnc$^{-/-}$ vs. WT animals (n = 4/4).

(E) Gene set enrichment analysis using the HALLMARK geneset collection. Enrichment plot for the fatty acid metabolism gene set using brown adipose tissue transcriptome data of mitolnc$^{-/-}$ vs. WT animals (n=4/4).

(F-H) BCAA levels in BAT tissue (F), liver tissue (G) and serum (H) (n = 9 WT, n = 8 mitolnc$^{-/-}$ for each tissue or serum)

(**I**) BCAA levels in serum (n = 12 WT, n = 12 mitolnc$^{-/-}$/mitolnc$^{pa/pa}$ serum samples using the Abcam BCAA assay)

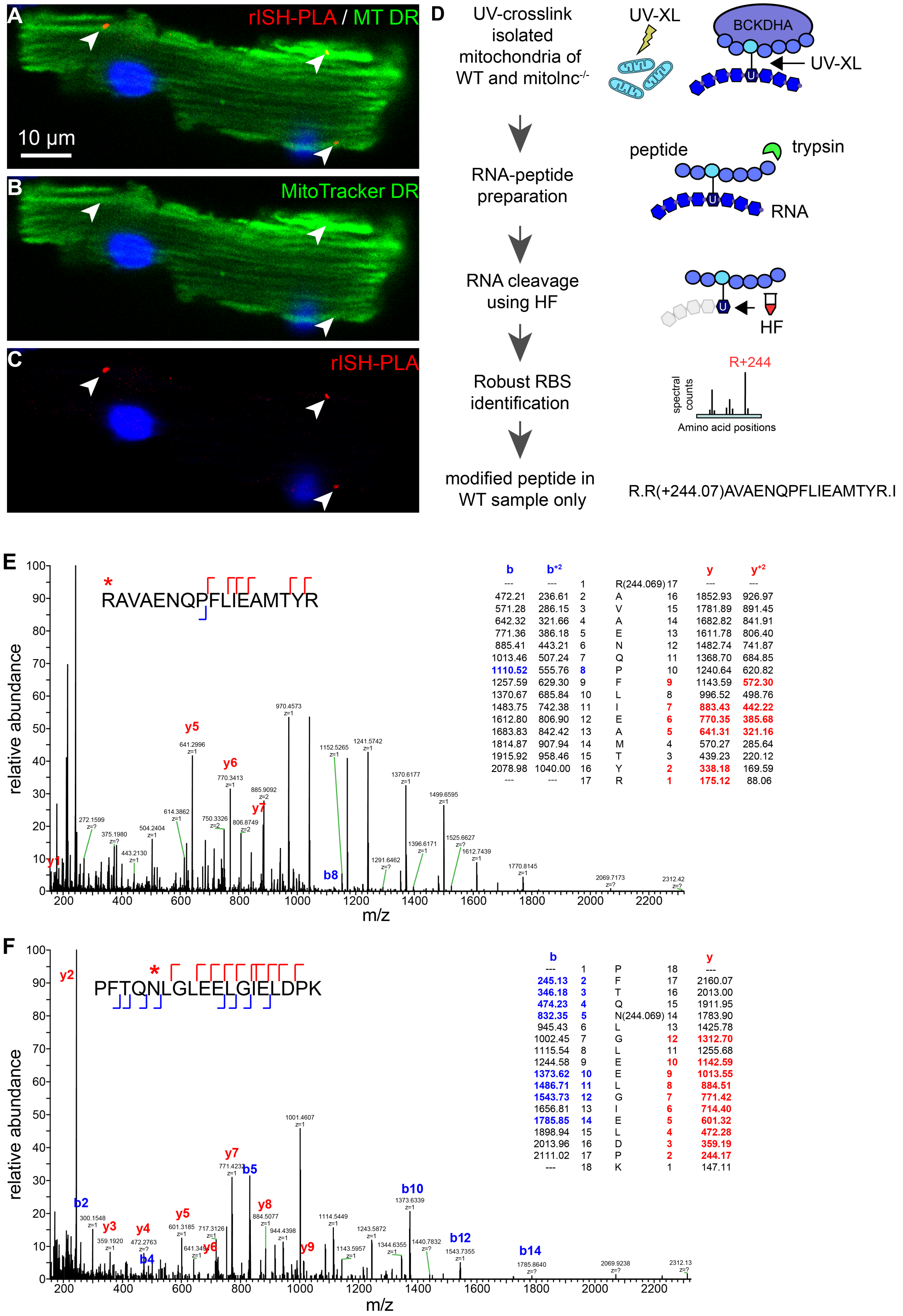
A
rISH-PLA / MT DR
10 µm
B
MitoTracker DR
C
rISH-PLA
D
UV-crosslink isolated mitochondria of WT and mitolnc-/-
UV-XL
BCKDHA
UV-XL
RNA-peptide preparation
peptide
trypsin
RNA
RNA cleavage using HF
HF
Robust RBS identification
R+244
spectral counts
Amino acid positions
modified peptide in WT sample only
R.R(+244.07)AVAENQPFLIEAMTYR.I
E
relative abundance
m/z
RAVAENQPFLIEAMTYR
b b+2 y y+2
--- --- 1 R(244.069) 17 --- ---
472.21 236.61 2 A 16 1852.93 926.97
571.28 286.15 3 V 15 1781.89 891.45
642.32 321.66 4 A 14 1682.82 841.91
771.36 386.18 5 E 13 1611.78 806.40
885.41 443.21 6 N 12 1482.74 741.87
1013.46 507.24 7 Q 11 1368.70 684.85
1110.52 555.76 8 P 10 1240.64 620.82
1257.59 629.30 9 F 9 1143.59 572.30
1370.67 685.84 10 L 8 996.52 498.76
1483.75 742.38 11 I 7 883.43 442.22
1612.80 806.90 12 E 6 770.35 385.68
1683.83 842.42 13 A 5 641.31 321.16
1814.87 907.94 14 M 4 570.27 285.64
1915.92 958.46 15 T 3 439.23 220.12
2078.98 1040.00 16 Y 2 338.18 169.59
--- --- 17 R 1 175.12 88.06
F
relative abundance
m/z
PFTQNLGLEELGIELDPK
b y
--- 1 P 18 ---
245.13 2 F 17 2160.07
346.18 3 T 16 2013.00
474.23 4 Q 15 1911.95
832.35 5 N(244.069) 14 1783.90
945.43 6 L 13 1425.78
1002.45 7 G 12 1312.70
1115.54 8 L 11 1255.68
1244.58 9 E 10 1142.59
1373.62 10 E 9 1013.55
1486.71 11 L 8 884.51
1543.73 12 G 7 771.42
1656.81 13 I 6 714.40
1785.85 14 E 5 601.32
1898.94 15 L 4 472.28
2013.96 16 D 3 359.19
2111.02 17 P 2 244.17
--- 18 K 1 147.11

Supplementary Figure S5: Interaction of mitolnc and BCKDH is mostly confined to mitochondria

(**A**) Confocal image of a cardiomyocyte with DAPI-stained nuclei (blue), fixed Mitotracker Deep Red (assigned color: green), and rISH-PLA signals (red). Arrowheads indicate rISH-PLA signals.

(**B**) Mitotracker (green) and DAPI (blue) channel of the image in A.

(**C**) DAPI (blue) and rISH-PLA signals (red) channel of the image in A.

(D) Schematic representation of mass spectrometry-based identification of RNA-protein interaction sites. RNA-protein interactions in mitochondria isolated from WT and mitolnc$^{-/-}$ hearts were crosslinked by UV treatment. Proteins were digested for mass spectrometry and phosphodiester-bonds in RNA molecules were cleaved by hydrofluoric acid. The hydrofluoric acid cleavage leaves a uridine of defined mass (244.07 Da) on peptides after crosslinking to RNA, allowing identification of modified peptides by MS. A modified peptide, originating from BCKDHA, was only detected in WT, but not in mitolnc$^{-/-}$ derived protein lysates, suggesting that the modification is caused by mitolnc-BCKDHA interactions. The peptide is located between the cofactor binding sites of BCKDHA and the phosphorylation sites and was deleted in the BCKDHA del5 mutant (adapted from Bae et al. 2020, Nat Struct Mol Biol; DOI: 10.1038/s41594-020-0436-2).

(E) Higher-energy collisional dissociation fragmentation spectra of triply charged modified ($C_9H_{12}N_2O_6$ = 244.069, uridine) peptide RAVAENQPFLIEAMTYR with labelled y (red) and b (blue) identification fragments, selected target ions indicated in the spectrum; this modified peptide was exclusively found in WT but not in KO.

(F) Higher-energy collisional dissociation fragmentation spectra of triply charged modified ($C_9H_{12}N_2O_6$ = 244.069, uridine) peptide PFTQNLGLEELGIELDPK with labelled y (red) and b (blue) identification fragments, selected target ions indicated in the spectrum; this modified peptide was found in WT and KO samples indicating additional interaction with a different RNA.

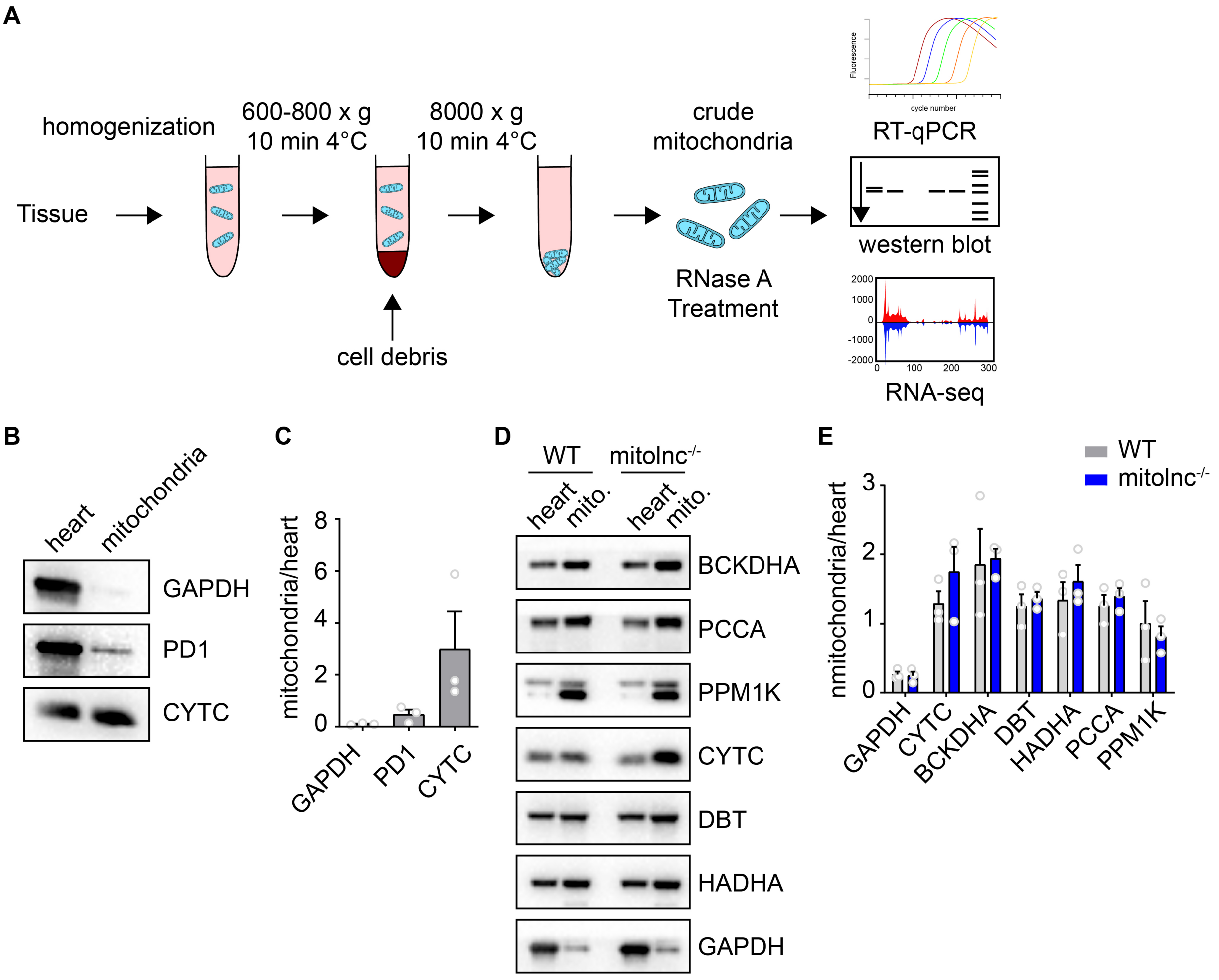

Supplementary Figure S6: Mitochondrial isolation does not reveal changes in the localization of components of BCAA catabolism.

(A) Scheme illustrating enrichment of mitochondria for analysis of mitochondrial proteins and RNA.

(B) Western blot analysis confirms the enrichment of mitochondrial proteins in WT heart samples after high speed centrifugation compared to whole heart lysates; Anti-GAPDH and anti-PD1 (Pdia3, protein disulfide isomerase associated 3) antibodies were used to confirm enrichment of mitochondria in contrast to protein localized in the cytoplasm or endoplasmic reticulum (n = 3 WT animals).

(C) Whole heart lysates and respective mitochondrial enrichment samples isolated from WT and mitolnc$^{-/-}$ hearts were analyzed for abundance of components of the BCAA catabolism

(D, E) Representative Western blots and statistical evaluation of whole heart lysates and mitochondrial enrichment samples (n = 3 WT, n = 3 mitolnc$^{-/-}$ hearts, two-way Anova, Fishers LSD test, no significant differences between whole heart lysates and respective mitochondrial enrichment samples)

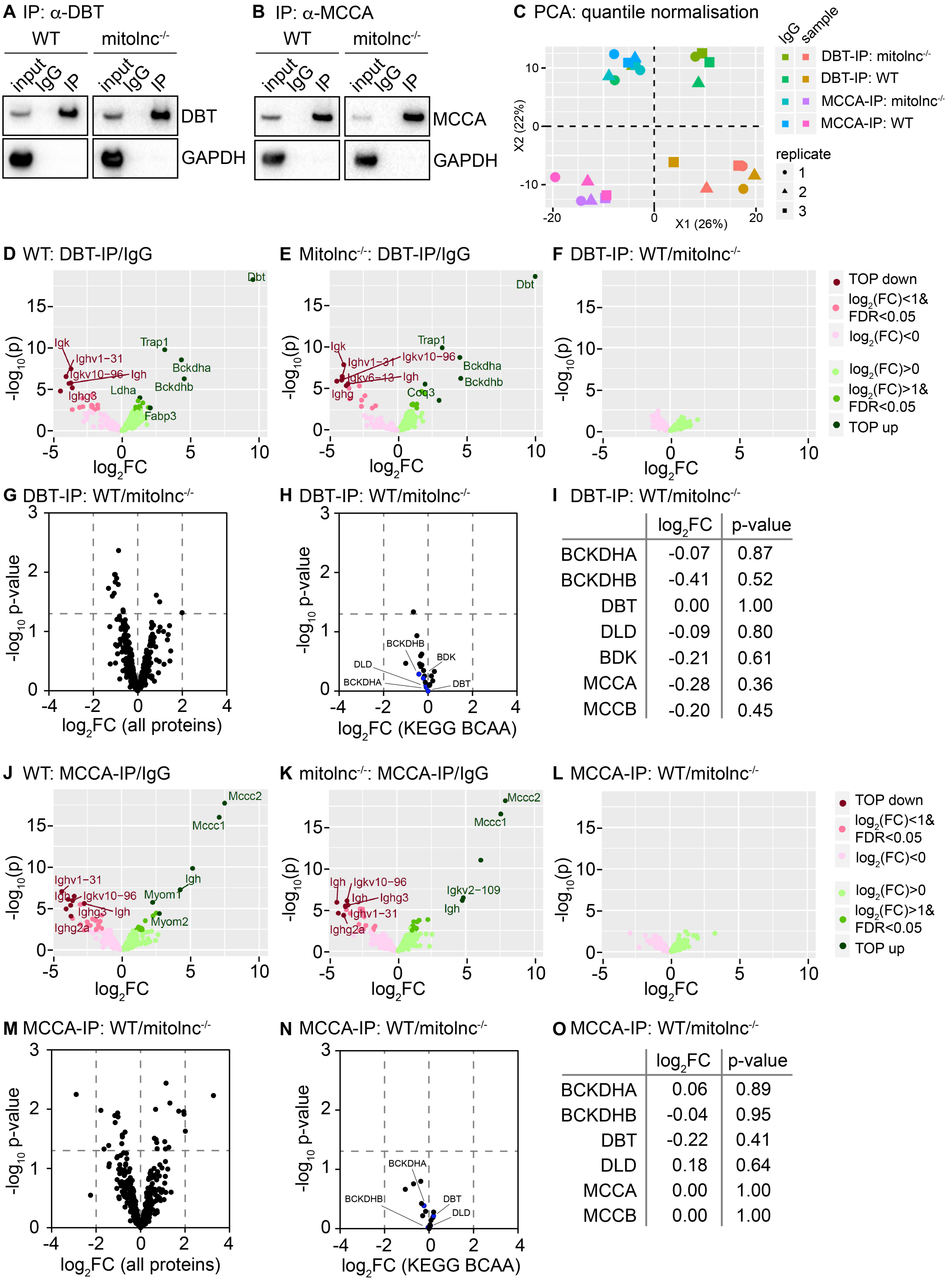

I DBT-IP: WT/mitolnc$^{-/-}$

	log_2FC	p-value
BCKDHA	-0.07	0.87
BCKDHB	-0.41	0.52
DBT	0.00	1.00
DLD	-0.09	0.80
BDK	-0.21	0.61
MCCA	-0.28	0.36
MCCB	-0.20	0.45

O MCCA-IP: WT/mitolnc$^{-/-}$

	log_2FC	p-value
BCKDHA	0.06	0.89
BCKDHB	-0.04	0.95
DBT	-0.22	0.41
DLD	0.18	0.64
MCCA	0.00	1.00
MCCB	0.00	1.00

Supplementary Figure S7: Co-immunoprecipitation of BCAA catabolic enzymes does not reveal changes in BCKDH complex composition upon deletion of mitolnc.

(A, B) Western blot to validate the immunoprecipitations (IPs) using antibodies directed against DBT or MCCA and protein lysates of WT and mitolnc$^{-/-}$ hearts.

(C) Principal component analysis of quantile normalized mass spectrometry data from IPs using antibodies directed against DBT or MCCA and protein lysates of WT and mitolnc$^{-/-}$ hearts (DBT: n = 3 WT, n = 3 mitolnc$^{-/-}$; MCCA: n = 3 WT, n = 3 mitolnc$^{-/-}$, specific antibody and IgG for each sample).

(D-H) Volcano plots of mass spectrometry data from IPs using protein lysates of WT and mitolnc$^{-/-}$ hearts and an antibody directed against DBT. (**D**, **E**) Immunoprecipitation vs. respective IgG controls, demonstrating significant enrichment of IP target proteins. (**F**) Volcano plot for IPs of WT vs. mitolinc$^{-/-}$ samples. No significant changes are apparent after FDR correction of p-values (n = 3 WT, n = 3 mitolnc$^{-/-}$; Bayesian–moderated t–testing; proteins significant after FDR correction (Benjamini-Hochberg) are labelled with dark colors). Volcano plot for IPs of WT vs. mitolinc$^{-/-}$ samples without FDR correction for (**G**) all detected proteins and for (**H**) proteins belonging to the KEGG BCCA degradation pathway (n = 3 WT, n = 3 mitolnc$^{-/-}$; Bayesian–moderated t–testing).

(I) Log_2 fold change (log_2FC) and p-values for BCKDH complex proteins obtained by mass spectrometry analysis of immunoprecipitates using antibodies directed against DBT. Protein lysates were either obtained from WT or mitolnc$^{-/-}$ hearts respectively (DBT: n = 3 WT, n = 3 mitolnc$^{-/-}$; Bayesian–moderated t–testing).

(J-N) Volcano plots of mass spectrometry data from IPs using protein lysates from WT and mitolnc$^{-/-}$ hearts and an antibody directed against MCCA. (**J**, **K**) Immunoprecipitation with MCCA antibodies vs. respective IgG controls demonstrate significant enrichment of the IP target proteins. (**L**) Volcano plot of mass spectrometry data from IPs of WT vs. mitolinc$^{-/-}$ samples after FDR correction of p-values (n = 3 WT, n = 3 mitolnc$^{-/-}$; Bayesian–moderated t–testing; proteins significant after FDR correction (Benjamini-Hochberg) are labelled with dark colors). Volcano plot of mass spectrometry data for IPs of WT vs. mitolinc$^{-/-}$ samples without FDR correction for (**M**) all detected proteins and for (**N**) detected proteins belonging to the KEGG BCCA degradation pathway (n = 3 WT, n = 3 mitolnc$^{-/-}$; Bayesian–moderated t–testing).

(O) Log_2 fold change (log_2FC) and p-values for BCKDH complex proteins obtained by mass spectrometry analysis of immunoprecipitates, using protein lysates of WT and mitolnc$^{-/-}$ hearts and antibodies directed against MCCA (MCCA: n = 3 WT, n = 3 mitolnc$^{-/-}$; Bayesian–moderated t–testing).
